# Essential and non-overlapping IL-2Rα-dependent processes for thymic development and peripheral homeostasis of regulatory T cells

**DOI:** 10.1038/s41467-019-08960-1

**Published:** 2019-03-04

**Authors:** Kevin H. Toomer, Jen Bon Lui, Norman H. Altman, Yuguang Ban, Xi Chen, Thomas R. Malek

**Affiliations:** 1 Department of Microbiology and Immunology, Miller School of Medicine, University of Miami, Miami, FL 33136 USA; 2 Department of Pathology, Miller School of Medicine, University of Miami, Miami, FL 33136 USA; 3 Sylvester Comprehensive Cancer Center, Miller School of Medicine, University of Miami, Miami, FL 33136 USA; 4 Department of Public Health Sciences, Miller School of Medicine, University of Miami, Miami, FL 33136 USA; 5 Diabetes Research Institute, Miller School of Medicine, University of Miami, Miami, FL 33136 USA

## Abstract

IL-2R signaling is essential for regulatory T cell (Treg) function. However, the precise contribution of IL-2 during Treg thymic development, peripheral homeostasis and lineage stability remains unclear. Here we show that IL-2R signaling is required by thymic Tregs at an early step for expansion and survival, and a later step for functional maturation. Using inducible, conditional deletion of CD25 in peripheral Tregs, we also find that IL-2R signaling is indispensable for Treg homeostasis, whereas Treg lineage stability is largely IL-2-independent. CD25 knockout peripheral Tregs have increased apoptosis, oxidative stress, signs of mitochondrial dysfunction, and reduced transcription of key enzymes of lipid and cholesterol biosynthetic pathways. A divergent IL-2R transcriptional signature is noted for thymic Tregs versus peripheral Tregs. These data indicate that IL-2R signaling in the thymus and the periphery leads to distinctive effects on Treg function, while peripheral Treg survival depends on a non-conventional mechanism of metabolic regulation.

## Abstract (web summary)

Interleukin-2 (IL-2) signaling is required for regulatory T (Treg) cell differentiation in the thymus, but its function in peripheral Tregs is still unclear. Here the authors show, using inducible deletion of IL-2 receptor subunit CD25, that IL-2 signaling is essential for maintaining peripheral Treg homeostasis, but dispensable for lineage stability.

## Introduction

IL-2R signaling is essential for regulatory T cells (Tregs) in part by driving activation of STAT5 that directly upregulates Foxp3 and CD25 in a positive feedback loop to establish and maintain Treg transcriptional identity^1–3^. Through this pathway, IL-2 promotes the maturation of CD4^+^ Foxp3^lo^ T cells into CD4^+^ CD25^+^ Foxp3^hi^ Tregs during thymic development^4–6^. Recent studies also point to a critical role for IL-2 in Treg function, as conditional ablation of IL-2Rα (CD25) or IL-2Rβ (CD122) in Tregs led to lethal autoimmunity^7^, similar to Foxp3-deficient scurfy mice^8^. Suppressive function was restored in these Tregs after expression of constitutively active STAT5^7^. Although these genetic tools have advanced our understanding of Treg function, they have not yet established the Treg-selective role of IL-2R signaling in the thymus, including the possibility of redundancy with IL-15 or inflammatory signals that are present in the context of autoimmune disease. Moreover, the extent to which accelerated disease is directly related to loss of Treg function, versus effects on thymic development or impaired IL-2 responsiveness of autoreactive T cells, has not yet been determined.

IL-2 also supports maintenance of Tregs in the periphery. However, the data corroborating this role are derived primarily from settings of immune reconstitution^9,10^, adoptive transfer^11^, or autoimmunity^12–14^ that may not reflect normal physiology. Blockade with anti-IL-2 monoclonal antibody (mAb) reduces abundance, physiological proliferation, and Foxp3 expression among Tregs early in life^15^, but only minimally affects the peripheral Treg compartment in adult mice^16^. Other studies have shown that signals through TCR^17–20^, CD28^21^, CTLA4^22^, TNF receptor superfamily (TNFRSF) members^23,24^, and IL-33^25,26^ contribute to peripheral Treg survival, expansion, and function. These data raise the possibility that IL-2 has a more limited role for Tregs post thymically. Furthermore, assessments of IL-2R signaling for Treg subsets suggest a complex and multifaceted role whereby IL-2 controls the survival of long-lived, resting CD62L^hi^ central Tregs (cTregs) as well as production of highly proliferative, terminally differentiated Klrg1^+-^activated effector Tregs (eTregs)^27,28^, while eTreg function appears to be enhanced by TCR and IL-2R signaling through a non-overlapping mechanism. Several studies have also suggested that IL-2 is essential to maintain the identity of peripheral Tregs^29,30^, but this issue has not been unequivocally addressed.

Since IL-2R signaling is critical during thymic Treg development, it has been difficult to establish the precise function of this pathway in Treg homeostasis and stability. This role cannot be ascertained using germline or Treg conditional knockout of IL-2/IL-2R because the targeted genes are ablated before or during thymic Treg development, obscuring true IL-2-dependent effects in mature Tregs. Here, we use a Treg CD25 conditional knockout (cKO) model to determine the role of IL-2R signaling during thymic development and for peripheral Tregs independent of the thymus, the latter by tamoxifen-induced CD25 deletion. Our approach is also designed to eliminate any confounding factors associated with the systemic autoimmunity typically caused by ablating IL-2R signaling in mice. Our study identifies overlapping but differential IL-2R-dependent functions for Treg thymic development and peripheral Treg homeostasis.

## Results

### Treg-targeted CD25^cKO^ produces a scurfy-like phenotype

Tregs selectively lacking expression of CD25 are nonfunctional, as mice with this defect rapidly succumb to lethal autoimmunity^7^. We produced CD25 conditional knockout (cKO) mice to assess the basis for this lethal disease and directly define the role of IL-2R signaling for Treg thymic development and peripheral homeostasis. In this model, C57BL/6 CD25^flox/flox^ mice were crossed to Foxp3^YFP/Cre^ mice so that resulting progeny expressed a nonfunctional *Il2ra* gene through Cre-mediated excision of exon 4 (Supplementary Fig. 1a, b). Tregs from these CD25^flox/Foxp3−YFP/Cre^ mice (designated CD25^cKO^) and mice with germline deletion of CD25 (designated CD25^gKO^) did not express CD25 (Supplementary Fig. 1c–e).

**Fig. 1:**
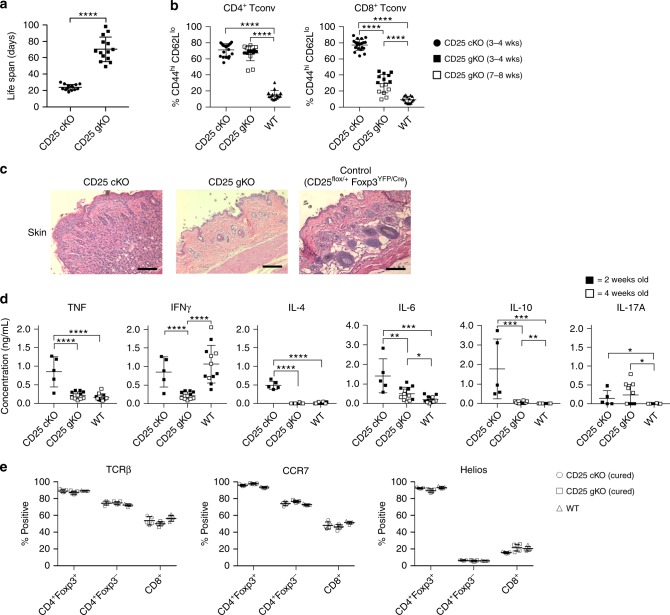
Lethal autoimmunity in CD25 Treg-targeted conditional and CD25 germline knockout mice. **a** Lethal autoimmune disease occurs in both CD25^flox/Foxp3-YFP/Cre^ mice (designated CD25^cKO^) and CD25^gKO^ mice, and is accelerated in the former. Life span refers to time until animals died spontaneously or developed clinical complications for which euthanasia was indicated (*n* = 14 mice per genotype). **b** Percentages of CD4^+^ and CD8^+^ T conventional (Tconv) cells from the indicated mice expressing a CD44^hi^ CD62L^lo^ phenotype, evaluated by ex vivo flow cytometry (*n* = 15–20 mice per genotype). **c** Representative H&E-stained dorsal skin sections from the indicated mice (magnification ×200, scale bar: 100 μm). **d** After stimulation with anti-CD3/CD28, cytokine production by splenocytes from the indicated mice was determined by bead capture assay (*n* = 5–12 mice per genotype). **e** Thymic selection based on expression of TCRβ, CCR7, and Helios was evaluated among single positive subsets from the indicated mice at 12–14 days of age (*n* = 5 mice per genotype). Each point represents an individual mouse (**a**, **b**, **d**, **e**; mean ± SD). *p < 0.05, **p < 0.01, ***p < 0.001, ****p < 0.0001 (two-tailed Student’s *t* test). Data are pooled from (**a**, **b**, **d**, **e**) three independent experiments

As expected, CD25^cKO^ and CD25^gKO^ mice exhibited lethal autoimmunity, but death for CD25^cKO^ mice occurred at a much younger age (3−4 weeks) (Fig. 1a). The disease process exhibited overlapping features in both knockout models, including autoimmune hemolytic anemia, weight loss, and lymphocytic infiltration of multiple organs (Supplementary Fig. 2b–f), with T conventional (Tconv) populations skewed toward a highly activated CD44^hi^ CD62L^lo^ phenotype (Fig. 1b). However, CD25^cKO^ mice uniquely demonstrated clinical and histological evidence of skin inflammation, including shrinking and crusting of the ears, scaling of the tail, squinting, and hair loss (Fig. 1c, Supplementary S2a). This abbreviated life span and skin involvement produced a phenotype similar to the scurfy (Foxp3-null) mouse, likely due in part to the absence of Treg function^8^.

CD25^gKO^ mice contained many fewer activated CD44^hi^ CD62L^lo^ CD8^+^ T cells than CD25^cKO^ mice. In addition, when splenocytes from these mice were stimulated with anti-CD3/CD28 in vitro, production of Th1 (TNF and IFNγ) and Th2 (IL-4, IL-6, and IL-10) cytokines was significantly greater in CD25^cKO^ mice. In contrast, IL-17 secretion was low, but elevated similarly, in T cells from CD25^cKO^ and CD25^gKO^ mice (Fig. 1d). These data indicate that T cells from CD25^cKO^ mice produce more Th1 and Th2 cytokines than CD25^gKO^ mice, and that these autoreactive T cells also appear earlier in life. Thus, the delayed disease in CD25^gKO^ mice likely reflects a loss of IL-2R function in autoreactive CD4^+^ and CD8^+^ T cells. Alternatively, the enhanced activity in peripheral autoreactive T cells in CD25^cKO^ mice might result from increased thymic production of self-reactive T cells due to the inability of developing Tregs to utilize IL-2. We explored this possibility by measuring surface markers on CD4^+^ and CD8^+^ single-positive (SP) thymocytes in order to interrogate the thymic selection processes. TCRβ is used as a surrogate for successful TCR gene rearrangement (β-selection). CCR7^31,32^ marks cells that have undergone successful positive selection. The transcription factor Helios discriminates thymocytes undergoing negative selection^33–35^.

For these and several experiments discussed below, CD25^cKO^ and CD25^gKO^ mice received wild-type (WT) splenocytes 1–2 days after birth to prevent development of autoimmunity (Supplementary Fig. 3a), an approach we previously used to prevent autoimmunity in IL-2Rβ-deficient mice^36^. These “cured” mice had normal life spans and no increase in the frequency of CD44^hi^ CD62L^lo^ activated T cells. Although total splenocytes were transferred, donor cells comprised ~40–60% of Tregs in the adult thymus and ~90–95% in the spleen, but only a negligible portion of Foxp3^−^ thymocytes and < 10% of Foxp3^−^ splenic T cells (Supplementary Fig. 3b–f). The finding that high selective engraftment of Tregs coincides with suppression of autoimmunity further corroborates the central role of IL-2R in Tregs for peripheral tolerance.

Overall percentages of SP, double-positive (DP), and double-negative (DN) thymocytes were identical between “cured” CD25^cKO^, “cured” CD25^gKO^, and WT littermate mice (Supplementary Fig. 4b). Expression of TCRβ, CCR7, and Helios among recipient-derived SP thymocytes from “cured” KO mice showed no significant deviations from WT, suggesting that β-selection, positive selection, and negative selection operate normally in the context of IL-2R deficiency (Fig. 1e). Thus, the functional inefficiency of Tconv cells in CD25^gKO^ mice is most likely a direct result of the absence of IL-2R signaling in the periphery rather than an effect within the thymus.

### IL-2R-dependent activities in thymic Treg development

**Fig. 2:**
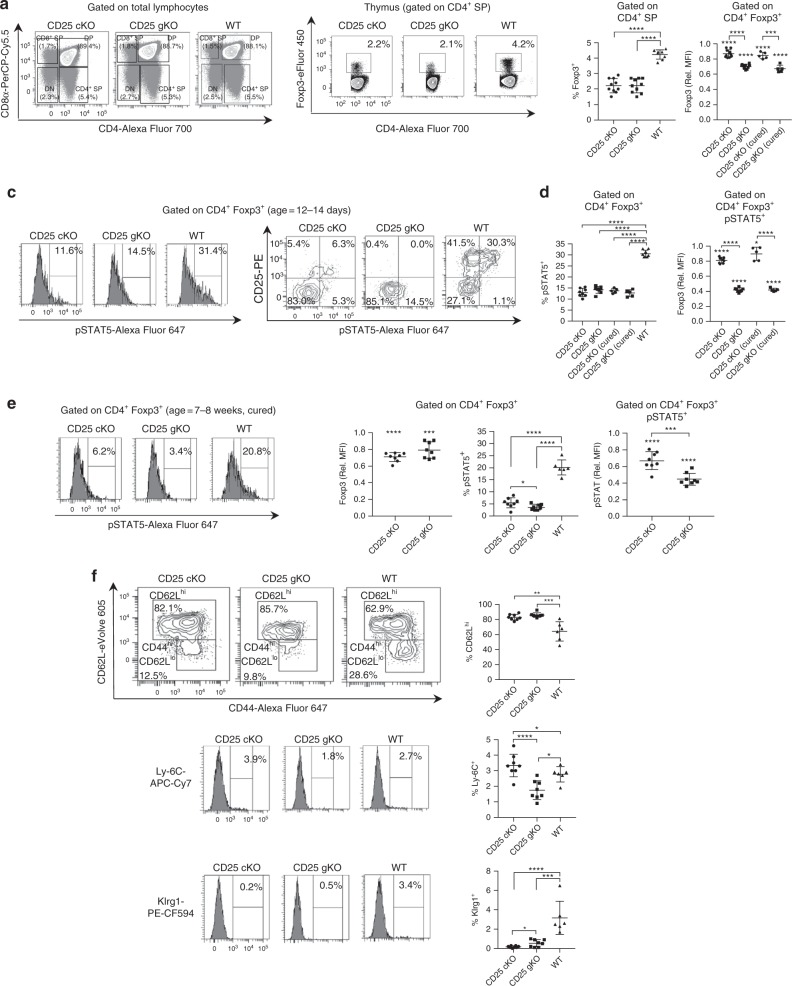
Properties of thymic Treg precursors in CD25^cKO^ and CD25^gKO^ mice. **a** Representative contour plots showing gating strategy for thymocyte subpopulations from CD25^flox/Foxp3-YFP/Cre^ (CD25^cKO^), CD25^gKO^, and WT mice. SP = single positive. DP double positive. DN double negative. **b** Representative contour plots showing Treg gating strategy among CD4^+^ SP thymocytes (left), with scatter plots for Foxp3 percentages and MFI (right). For MFI measurements, mice “cured” of autoimmunity through adoptive transfer of WT splenocytes were also evaluated (age = 12–14 days; *n* = 5–10 mice per group). **c** Representative histograms, contour plots, and gating strategy showing STAT5 phosphorylation and CD25 expression among CD4^+^ Foxp3^+^ thymocytes measured by ex vivo flow cytometry (age = 12–14 days). **d** Percentages and MFI for pSTAT5 among CD4^+^ Foxp3^+^ thymocytes (age = 12–14 days; *n* = 5–8 mice per group). **e** Representative histograms and scatter plots for Foxp3 levels (based on MFI) and pSTAT5 for host-derived CD4^+^ Foxp3^+^ thymocytes from “cured” adult mice (age = 7–8 weeks; *n* = 6–8 mice per group). **f** Representative contour plots/histograms (left) and scatter plots (right) showing expression of the cTreg/eTreg differentiation markers CD44, CD62L, Ly-6C, and Klrg1 among host-derived CD4^+^ Foxp3^+^ thymocytes from “cured” adult mice (age = 7–8 weeks; *n* = 6–8 mice per group). Rel. MFI = relative MFI normalized to CD25 WT controls. Each symbol represents an individual mouse (**b**, **d**, **e**, **f**; mean ± SD). *p < 0.05, **p < 0.01, ***p < 0.001, ****p < 0.0001. Symbols located directly above a single group of dots represent the comparison of that group against normalized WT control values (one sample *t* test); symbols located above brackets connecting two groups of dots represent inter-group comparisons (two-tailed Student’s *t* test). Data are representative of (**a**, **b**, **c**, **e**, **f**) or pooled from (**b**, **d**, **e**, **f**) three independent experiments

Thymic Treg development was compared among CD25^cKO^, CD25^gKO^, and WT mice at 12–14 days of age. Despite proportions of SP thymocytes comparable with WT (Fig. 2a), CD4^+^ Foxp3^+^ cells were reduced by 50% in CD25^cKO^ and CD25^gKO^ mice. When evaluated by mean fluorescence intensity (MFI) normalized to WT controls, Foxp3 levels within this population were reduced by 15 and 30% in CD25^cKO^ and CD25^gKO^ mice, respectively (Fig. 2b). The proportion of pSTAT5^+^ cells was similarly reduced for CD25^cKO^ and CD25^gKO^ Foxp3^+^ thymocytes (Fig. 2c, d, left panels), although lower pSTAT5 levels were observed in CD25^gKO^ mice when measured by normalized MFI, a pattern which was identical in recipient cells from “cured” mice (Fig. 2d, right panel). This pSTAT5 was associated with Foxp3^+^ CD25^+^ cells from WT and CD25^cKO^ mice, albeit on a lower fraction of cells for the latter (Fig. 2c, right panel), a result consistent with IL-2-dependent STAT5 activation in these developing Tregs. In contrast, Foxp3^+^ pSTAT5^+^ thymocytes from CD25^gKO^ mice were exclusively CD25^neg^ pSTAT5^lo^. Although CD25^gKO^ mice showed greater Foxp3 reduction than CD25^cKO^ mice at 2 weeks of age (Fig. 2b), “cured” CD25 knockout models did not show this difference at 7–8 weeks of age (Fig. 2e). Recipient-derived Foxp3^+^ cells in these “cured” adult CD25^cKO^ and CD25^gKO^ mice showed a lower proportion of pSTAT5^+^ cells than younger animals (Fig. 2e). These findings are consistent with redundant STAT5 phosphorylation in the developing Treg compartment in CD25^gKO^ mice by IL-7, IL-15, or other STAT5-activating cytokines^37–40^ that is more pronounced early in life. Examination of Foxp3^+^ thymocytes from these mice revealed that CD62L^hi^ cTregs predominated in WT mice, but were even more abundant in CD25^cKO^ and CD25^gKO^ mice (Fig. 2f), suggesting that IL-2R signaling contributes to development of thymic Tregs with a more activated phenotype.

**Fig. 3:**
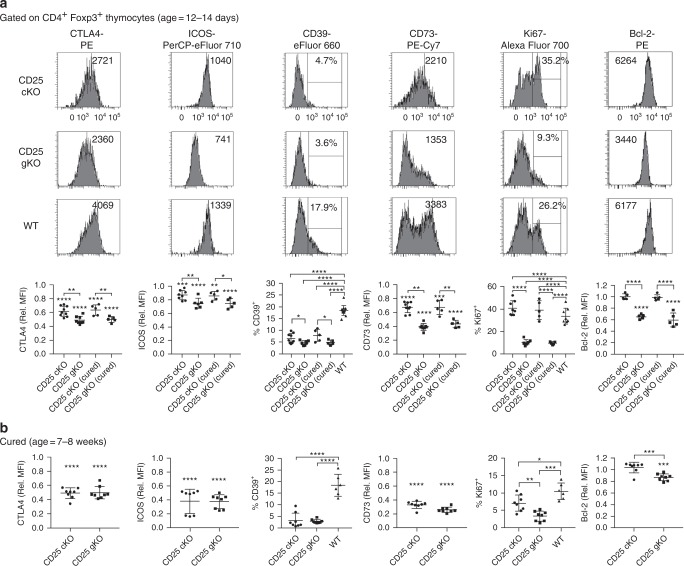
Abnormal programming of thymic Treg precursors in CD25^cKO^ and CD25^gKO^ mice. **a** Representative histograms (upper panels) and scatter plots (lower panels) showing expression of indicated markers, gated on CD4^+^ Foxp3^+^ thymocytes from CD25^flox/Foxp3-YFP/Cre^ (CD25^cKO^) and CD25^gKO^ mice (age = 12–14 days; *n* = 5–13 mice per group). **b** The indicated markers in (**a**) were evaluated in host-derived CD4^+^ Foxp3^+^ thymocytes from “cured” adult CD25^cKO^ and CD25^gKO^ mice (age = 7–8 weeks; *n* = 6–8 mice per group). Rel. MFI = relative MFI normalized to CD25 WT controls. Each symbol represents an individual mouse (**a**, **b**; mean ± SD). *p < 0.05, **p < 0.01, ***p < 0.001, ****p < 0.0001. Symbols located directly above a single group of dots represent the comparison of that group against normalized WT control values (one sample *t* test); symbols located above brackets connecting two groups of dots represent inter-group comparisons (two-tailed Student’s *t* test). Data are representative of (**a**) or pooled from (**a**, **b**) four independent experiments

These results suggest that thymic Treg development is more severely impaired in CD25^gKO^ mice. Accordingly, CD4^+^ Foxp3^+^ thymocytes in CD25^cKO^ and CD25^gKO^ mice at 12–14 days of age expressed lower levels of the key Treg functional markers CTLA4, ICOS, CD39, and CD73, a change which was more pronounced in the CD25^gKO^ model (Fig. 3a). These deficits persisted in recipient-derived cells from “cured” mice at 7–8 weeks of age, although the magnitude of reduction in adults was comparable between the two knockout models (Fig. 3b). CD25^gKO^ mice also showed unique deficits in Ki67 and Bcl-2 expression, which persisted into adulthood (Fig. 3a, b). Despite the more severe impact on thymic development in CD25^gKO^ mice, the CD25^cKO^ phenotype also points to a critical and late IL-2R-dependent programming step during Treg maturation, as well as a role for IL-2R signaling in autoreactive T cells, which accounts for the more severe autoimmunity in this model.

### CD25 KO mice retain numerous peripheral CD4^+^ Foxp3^+^ T cells

**Fig. 4:**
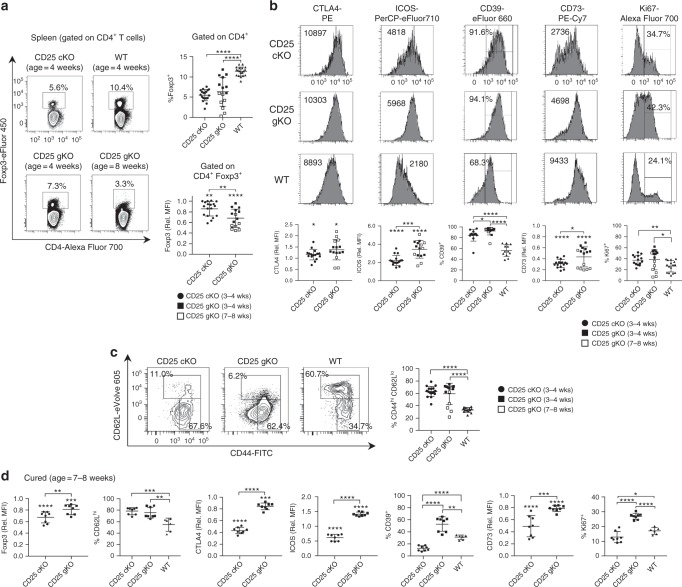
Splenic CD4^+^ Foxp3^+^ T cells from CD25^cKO^ and CD25^gKO^ mice are skewed toward eTregs. **a** Representative contour plots (left) showing Foxp3 expression and gating strategy in splenic CD4^+^ T cells, with scatter plots (right) displaying Foxp3 expression as a proportion of total splenic CD4^+^ T cells and by mean fluorescence intensity (MFI) at the specified ages (*n* = 7–19 mice per group). **b** The indicated markers were evaluated in splenic CD4^+^ Foxp3^+^ T cells from CD25^cKO^, CD25^gKO^, and WT mice at the specified ages by ex vivo flow cytometry. Representative histograms (upper panels) and scatter plots (lower panels) are shown (*n* = 7–16 mice per group). **c** Representative contour plots (left) and scatter plot (right) showing the proportion of splenic CD4^+^ Foxp3^+^ T cells with a CD44^hi^ CD62L^lo^ eTreg phenotype in CD25^cKO^, CD25^gKO^, and WT mice at the specified ages (*n* = 7–17 mice per group). **d** Scatter plots showing expression of the indicated markers for host-derived splenic CD4^+^ Foxp3^+^ T cells from ‘cured’ mice (age 7–8 weeks; *n* = 6–8 mice per group). Rel. MFI = relative MFI normalized to CD25 WT controls. Each symbol represents an individual mouse (**a**, **b**, **c**, **d**; mean ± SD). *p < 0.05; **p < 0.01; ***p < 0.001; ****p < 0.0001. Symbols located directly above a single group of dots represent the comparison of that group against normalized WT control values (one sample *t* test); symbols located above brackets connecting two groups of dots represent inter-group comparisons (two-tailed Student’s *t* test). Data are representative of (**a**, **b**, **c**) or pooled from (**a**, **b**, **c**, **d**) three independent experiments

In the setting of autoimmunity, the periphery of 3–4-week-old CD25^cKO^ and CD25^gKO^ mice contained a substantial proportion of CD4^+^ Foxp3^+^ T cells, albeit diminished in comparison with littermate controls. Similar to the thymus, expression of Foxp3 and CD73 in splenic CD4^+^ Foxp3^+^ T cells from these knockout mice was lower than WT (Fig. 4a, b). However, in striking contrast to the thymus, these cells were dominated by a CD44^hi^ CD62L^lo^ eTreg phenotype, rather than a cTreg phenotype (Fig. 4c) and expressed elevated levels of CTLA4, ICOS, and CD39 (Fig. 4b). Ki67 expression was largely comparable with WT Tregs, consistent with the ability of this population to readily proliferate in vivo (Fig. 4b). Thus, failure of these CD4^+^ Foxp3^+^ T cells to suppress autoimmunity in CD25^cKO^ mice cannot be attributed to their absence in the periphery, but is instead consistent with a lack of suppressive function.

When CD25^cKO^ and CD25^gKO^ mice were “cured” of autoimmunity through adoptive transfer of WT splenocytes, donor Tregs dominated the periphery. The failure of recipient-derived CD4^+^ Foxp3^+^ T cells from the knockout models to compete with WT Tregs further illustrates that CD4^+^ Foxp3^+^ T cells that develop without IL-2R signaling are defective. In the absence of autoimmunity, the phenotype of recipient-derived CD4^+^ Foxp3^+^ T cells from CD25^cKO^ mice largely resembled the immature Tregs from their thymus. Thus, in comparison with WT Tregs, the levels of not only CD73 but also CTLA4, ICOS, CD39, and Ki67 were reduced, and these cells were skewed toward a cTreg phenotype (Fig. 4d). Thus, the vigorous scurfy-like autoimmunity associated with CD25^cKO^ mice appears to activate peripheral CD4^+^ Foxp3^+^ T cells, but these changes fail to prevent lethal disease.

### CD25 KO Tregs are gradually lost from peripheral tissues

The essential action of IL-2R signaling for Treg development severely limits the utility of CD25^cKO^ and CD25^gKO^ mice for studying contributions of IL-2 to Treg homeostasis in peripheral tissues. To avoid this problem, we selectively targeted CD25 in peripheral Tregs in adult mice. Foxp3^eGFP-Cre-ERT2^ harbors a fusion protein combining enhanced green fluorescent protein (eGFP), Cre recombinase, and mutated human estrogen receptor ligand-binding domain (ERT2) that is expressed from the *Foxp3* locus. When these mice are crossed to R26^flox/stop/YFP^ (R26Y) mice, tamoxifen administration triggers nuclear localization of the fusion protein, inducing constitutive, heritable cell labeling with eYFP^16^. By further crossing our CD25^flox/flox^ allele with this reporter strain, mice were obtained, designated CD25^flox/Foxp3eGFP-Cre-ERT2/R26Y^, to abrogate CD25 expression on mature Tregs in vivo and track their persistence and phenotype over time (Supplementary Fig. 5).

**Fig. 5:**
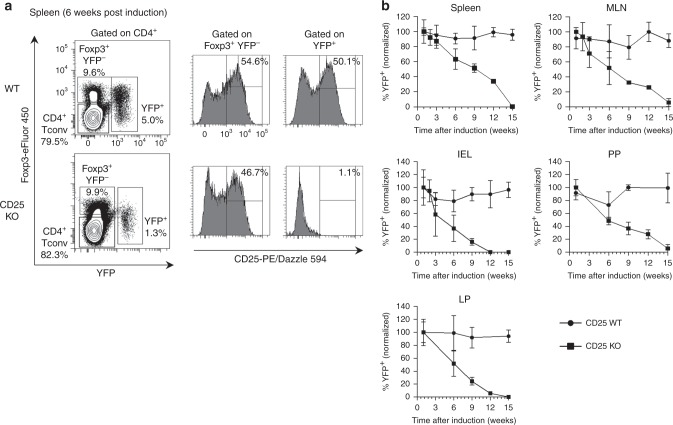
Impaired peripheral homeostasis of Tregs with selective CD25 deletion. **a** Representative contour plots and histograms showing splenocytes from CD25^flox/Foxp3eGFP−Cre-ERT2/R26Y^ mice (shown as CD25 KO) and reporter controls, analyzed 6 weeks after tamoxifen induction. **b** Proportional abundance of YFP^+^ cells in lymphoid and non-lymphoid tissues analyzed at indicated time points after tamoxifen induction. YFP^+^ cell numbers were calculated as a percentage of total Tregs (the combined Foxp3^+^ and YFP^+^ populations) and normalized to an initial induction efficiency of 100% measured 1 week after tamoxifen induction. MLN mesenteric lymph nodes, PP Peyer’s patches, IEL intraepithelial lymphocytes of small intestine, LP  lamina propria of small intestine (*n* = 3 mice per time point; error bars ± SD). Data are representative of (**a**) or pooled from (**b**) three independent experiments

Technical obstacles related to the dimness of the GFP reporter and spectral overlap between GFP and YFP prevented reliable discrimination between these two markers. To circumvent this issue, a modified intracellular flow-cytometry protocol was used to detect Foxp3 via antibody staining, with simultaneous retention of YFP signal. As expected, CD25 was not detected on YFP^+^ cells from CD25^flox/Foxp3eGFP-Cre-ERT2/R26Y^ mice (Fig. 5a). On this basis, the size of the YFP^+^ Treg population was calculated as a proportion of total Tregs, providing an estimate of induction efficiency and a means to control for inter-individual variations in Treg abundance.

The persistence of YFP-labeled cells was tracked over time in CD25^flox/Foxp3eGFP-Cre-ERT2/R26Y^ mice and reporter controls. YFP-labeled cells in CD25^flox/Foxp3eGFP-Cre-ERT2/R26Y^ mice gradually declined in frequency in all tissues examined, falling below the threshold of detection at ~12 weeks post induction for the small intestinal intraepithelial lymphocytes (IELs) and lamina propria (LP) lymphocytes and 15 weeks post induction for the spleen, mesenteric lymph nodes (MLN), and Peyer’s patches (PP). YFP-labeled cells from reporter controls, in contrast, remained at a stable frequency throughout this time period (Fig. 5b). Thus, a threshold level of IL-2 signaling appears to be essential for homeostatic maintenance of mature Tregs in peripheral lymphoid and non-lymphoid tissues.

### Site-specific transcriptional signatures of CD25 KO Tregs

**Fig. 6:**
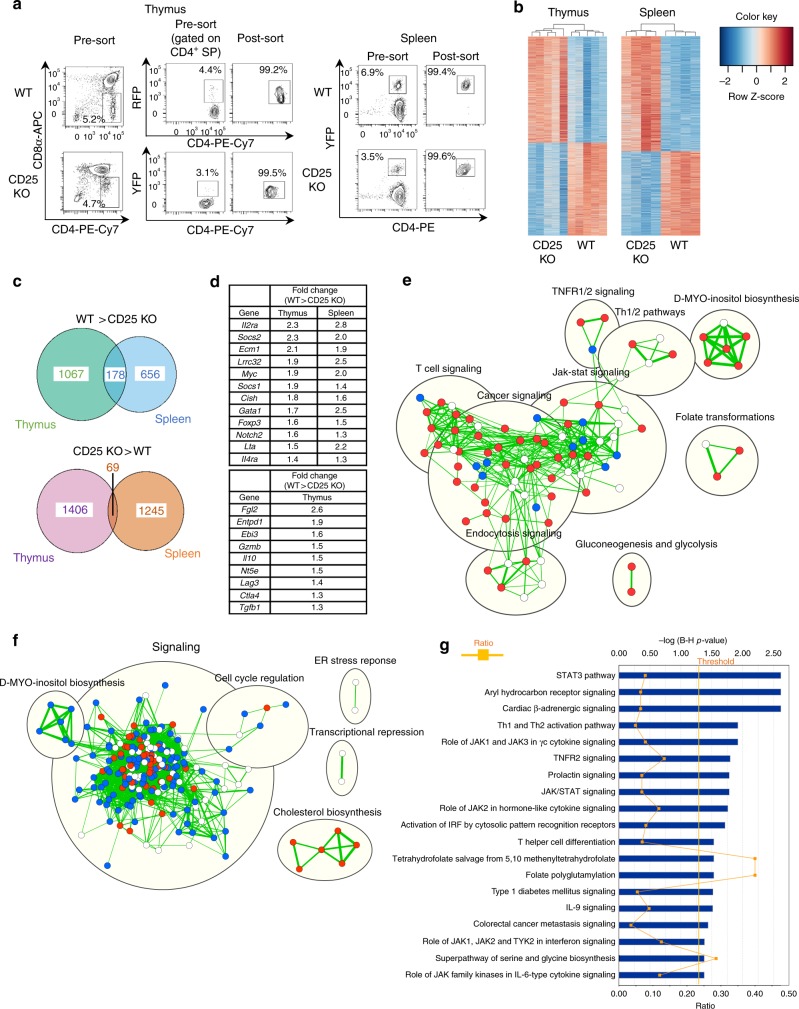
Distinctive transcriptional signatures in CD25-deficient thymic and peripheral Tregs. **a** Sorting strategy and purity of Foxp3^+^ Tregs FACS-purified from the thymus and spleen. Thymic Tregs were obtained from “cured” CD25^flox/Foxp3-YFP/Cre^ (CD25^cKO^) mice at 3–4 weeks of age, with age-matched Foxp3/RFP reporter mice used as controls. Splenic Tregs were obtained from CD25^flox/Foxp3eGFP−Cre-ERT2/R26Y^ mice and reporter controls 10–14 days after tamoxifen induction. **b** Set of differentially expressed (DE) transcripts in RNA-seq analysis of CD25 KO Tregs in thymus and spleen. DE transcripts were designated using a significance cutoff of false discovery rate (FDR) < 0.01, with each column representing an individual mouse (*n* = 5 for thymus; *n* = 4 for the spleen). **c** Venn diagrams showing overlap of genes from thymus and spleen that were downregulated (top) or upregulated (bottom) in CD25 KO Tregs. **d** Top: selected genes with reduced expression in CD25 KO Tregs from both thymus and spleen (FDR < 0.01 for all comparisons). Bottom: selected genes with reduced expression in CD25 KO Tregs from thymus only (FDR < 0.05 for all comparisons). **e** Ingenuity Pathway Analysis (IPA) pathway enrichment map for thymus. **f** IPA pathway enrichment map for the spleen. For pathway enrichment maps, pathways upregulated in CD25 KO Tregs are shown in blue, those downregulated in CD25 KO Tregs are shown in red, and pathways enriched without known direction are shown in white. Cutoffs for pathway selection were FDR < 0.1 for the thymus and FDR < 0.01 for the spleen, with *p*-value < 0.05 for both. **g** IPA conducted on DE genes with overlapping changes in the same direction for thymus and spleen. Pathways are listed in order of statistical significance, quantified on the top *x*-axis as the −log of *p-*values adjusted by the Benjamini–Hochberg (B-H) procedure. Cutoff for statistical significance is designated by the vertical line labeled “threshold.” “Ratio” values, quantified on the bottom *x*-axis, were calculated for each pathway by dividing the number of statistically significant genes in the data set by the total number of genes in the pathway

To determine IL-2Rα-dependent gene programs operative in thymic and peripheral Tregs, RNA-seq was performed on FACS-purified, CD25-deficient Tregs and reporter controls from the thymus of “cured” CD25^cKO^ mice and the spleen of tamoxifen-induced CD25^flox/Foxp3eGFP-Cre-ERT2/R26Y^ mice, to eliminate any confounding effects of autoimmunity (Fig. 6a). In total, 2718 differentially expressed (DE) transcripts were associated with thymic Tregs, and 2156 DE transcripts were found in splenic Tregs (Fig. 6b). In total, 178 DE transcripts with greater expression in WT Tregs were shared between the thymus and spleen, but many fewer were shared when considering transcripts that were more abundant in CD25 KO Tregs (Fig. 6c). Representative shared transcripts include the well-known IL-2-dependent genes *Foxp3*, *Myc*, *Cish*, *Socs1*, *Socs2*, and *Lta* (Fig. 6d, upper group). Furthermore, many mRNAs encoding molecules associated with Treg suppressive function were reduced in thymic CD25-deficient CD4^+^ Foxp3^+^ T cells (Fig. 6d, lower group), consistent with impaired functional programming. *Il2ra* transcripts were found to be reduced, rather than absent, in CD25 KO Tregs. The absence of exon 4 in these transcripts confirmed the lack of functional CD25 expression, as illustrated for peripheral Tregs (Supplementary Fig. 6).

Ingenuity pathway analysis (IPA) was conducted for both the spleen and thymus RNA-seq data sets. The three largest groups of IL-2-driven genes in the thymus were related to cancer, Jak/STAT, and T cell signaling, consistent with developmental progression of Tregs. The analysis also suggested that IL-2 promotes glycolysis among developing thymic Tregs (Fig. 6e). When compared with thymic Tregs, IL-2 appears to play a very distinctive role in the periphery. Cholesterol biosynthesis represented the only process that was normally IL-2R-dependent, suggesting that lipid metabolism may support oxidative phosphorylation for IL-2-driven homeostasis of Tregs. In contrast, a large number of interconnected pathways related to signaling and cell-cycle progression were associated with CD25-deficient Tregs, raising the possibility that IL-2R signaling in peripheral Tregs acts to dominantly repress gene activation (Fig. 6f). Overall, these data demonstrate largely unique IL-2R-dependent gene expression for thymic and peripheral Tregs, especially for mRNAs downregulated in response to IL-2.

Many pathways that overlapped between thymic and peripheral Tregs represent cytokine and Jak/STAT signaling, consistent with an expected common role for proximal IL-2R signaling in Tregs. Several other shared pathways (tetrahydrofolate salvage from 5,10 methenyltetrahydrofolate, folate polyglutamylation, and superpathway of serine and glycine biosynthesis) indicate a role for IL-2R signaling in amino acid and nucleotide biosynthesis. Th1 and Th2 pathways were also identified as IL-2R-dependent in both Treg compartments (Fig. 6g). For the thymus, IL-2R-dependent genes identified in these pathways did not include Th1 or Th2 cytokines, but rather several transcriptional regulators and immunological mediators that might act to promote development into functional suppressor cells specialized to inhibit Th1 and Th2 responses. However, transcripts for several effector cytokines (*Ifng*, *Il4*, and *Il10*) were increased in CD25-deficient peripheral Tregs (Supplementary Fig. 7). One surprising result in the analysis of Th1 and Th2 pathway constituents is that many were differentially expressed in opposite directions, suggesting that the IL-2 gene signature differs for thymic versus peripheral Tregs. However, we cannot exclude the possibility that these differences simply reflect the fact that, in the absence of IL-2R signaling, thymic Tregs are skewed toward a cTreg phenotype (Fig. 2f) whereas peripheral Tregs are skewed toward an eTreg phenotype (see below).

**Fig. 7:**
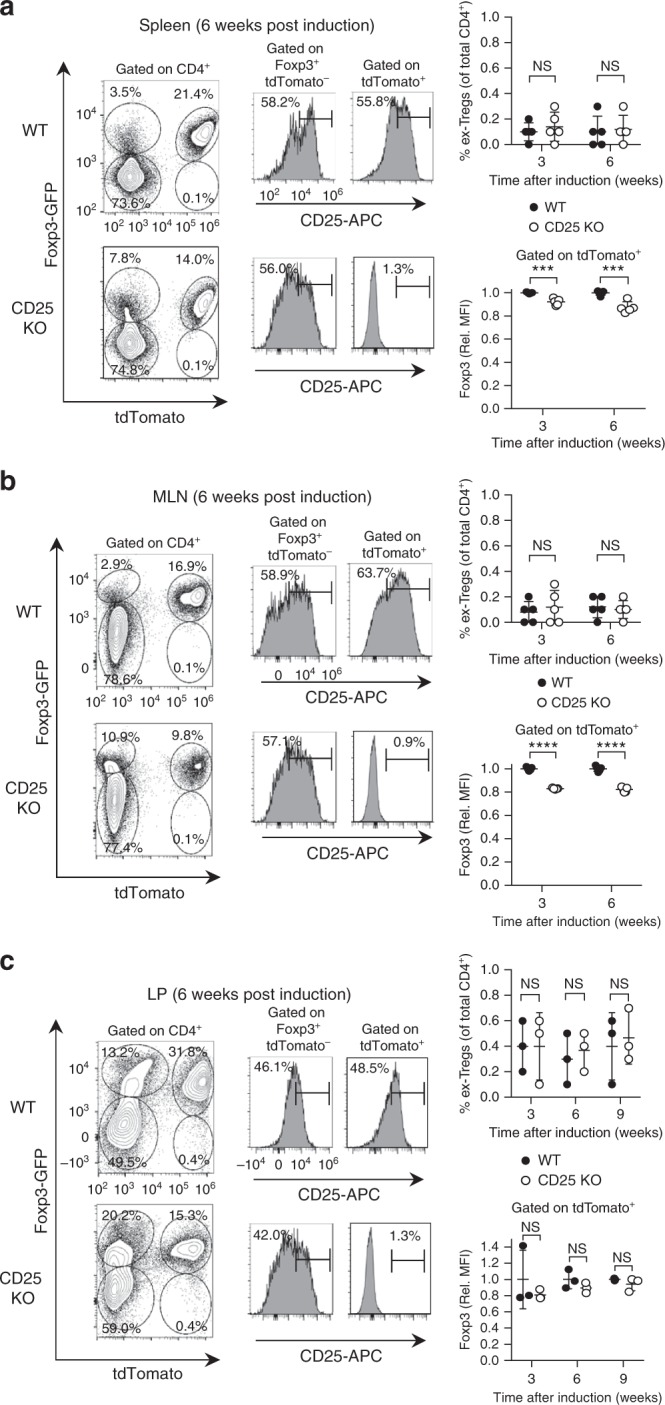
CD25 KO peripheral Tregs show only a modest decline in Foxp3 expression. Expression of GFP, tdTomato, and CD25 was evaluated by flow cytometry in the (**a**) splenic, (**b**) mesenteric lymph node (MLN), and (**c**) small intestinal lamina propria (LP) CD4^+^ T cells from CD25^flox/Foxp3eGFP-Cre-ERT2/R26TD^ mice (shown as CD25 KO) and reporter controls. Representative contour plots and histograms are shown (left panels). Scatter plots display the proportion of GFP^-^ tdTomato^+^ “ex-Tregs” in each tissue, expressed as a percentage of total CD4^+^ T cells (upper right panels), as well as GFP MFI among tdTomato^+^ Tregs from CD25^flox/Foxp3eGFP-Cre-ERT2/R26TD^ mice and reporter controls (lower right panels). These parameters were evaluated in the spleen and MLN at 3 and 6 weeks post induction (*n* = 5 mice per group). In LP, the proportion of GFP^−^ tdTomato^+^ “ex-Tregs” and GFP MFI among tdTomato^+^ Tregs were examined at 3, 6, and 9 weeks post induction (*n* = 3 mice per group). Rel. MFI = relative MFI normalized to CD25 WT controls. Each symbol represents an individual mouse (**a**, **b**, **c**; mean ± SD). NS, not significant (*p* > 0.05), ***p < 0.001, ****p < 0.0001 (two-tailed Student’s *t* test). Data are representative of, or pooled from (**a**, **b**, **c**) three independent experiments

The presence of transcripts for signature Th1 and Th2 cytokines in CD25 KO peripheral Tregs, and the detection of apparent Foxp3^−^ YFP^+^ cells in the spleen of tamoxifen-induced mice (Fig. 5a), raised the possibility that IL-2R signaling deficiency among Tregs in the periphery may support plasticity toward generation of stable CD4^+^ T effector cells. However, this result might simply reflect suboptimal discrimination of Foxp3^+^ and Foxp3^−^ cells due to the permeabilization conditions used to preserve YFP fluorescence. To clarify the role of IL-2 in maintaining Treg stability, a Rosa26^stop^ mouse with a tdTomato reporter (R26TD) was used to replace YFP in conjunction with CD25^flox/Foxp3eGFP-Cre-ERT2^ (referred to as CD25^flox/Foxp3eGFP-Cre-ERT2/R26TD^) to generate tdTomato-labeled peripheral Tregs that lacked CD25. At 3–9 weeks post induction, only a small population of CD4^+^ Foxp3^−^ tdTomato^+^ T cells (~0.1–0.7%) was detected in the spleen (Fig. 7a), MLN (Fig. 7b), or small intestine lamina propria (Fig. 7c) from reporter control mice, and these “ex-Tregs” did not increase in mice whose Tregs lacked CD25. Normalized GFP MFI values from tdTomato-labeled Tregs revealed a statistically significant but quantitatively modest (<20%) decline in Foxp3 expression among CD25 KO Tregs from the spleen (Fig. 7a) and MLN (Fig. 7b) relative to CD25 WT reporter controls when examined 3–6 weeks post induction. Of note, examination of GFP MFI values in the small intestine lamina propria revealed no statistically significant difference between tdTomato-labeled CD25 KO Tregs and CD25 WT reporter controls at 3, 6, or 9 weeks post induction (Fig. 7c). These data indicate that chronic lack of IL-2R signaling in peripheral Tregs does not support the development of a substantial stable pool of CD4^+^ Tconv cells, even in the gut mucosa.

### CD25 KO peripheral Tregs exhibit polarization toward eTregs

**Fig. 8:**
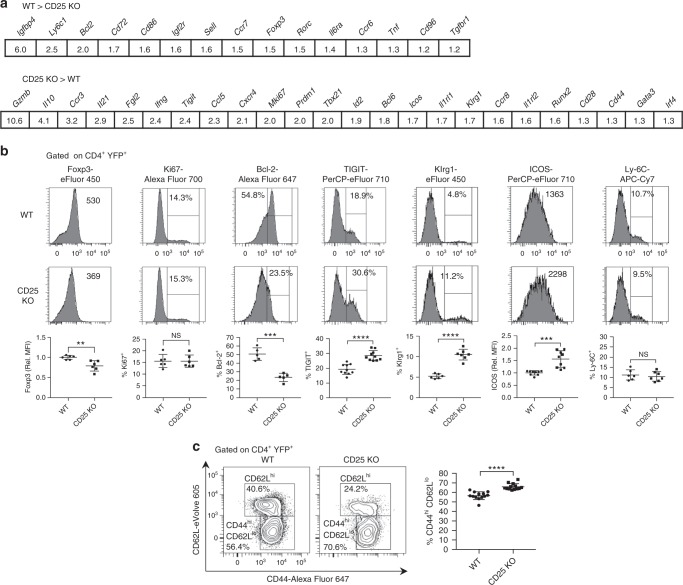
CD25 deficiency in peripheral Tregs initially favors survival of eTregs. **a** Selected immunological genes downregulated (top) or upregulated (bottom) in splenic CD25 KO Tregs from tamoxifen-induced CD25^flox/Foxp3eGFP-Cre-ERT2/R26Y^ mice evaluated by RNA-seq. Statistical significance is FDR < 0.01 for all comparisons. **b** Expression of indicated markers in splenic YFP^+^ Tregs from CD25^flox/Foxp3eGFP-Cre-ERT2/R26Y^ mice (shown as CD25 KO) and reporter controls was measured by ex vivo flow cytometry 10–14 days after tamoxifen induction (*n* = 5–10 mice per group). Representative histograms (upper panels) and scatter plots (lower panels) are shown. **c** Representative contour plots (left) and scatter plot (right) showing the proportion of splenic YFP^+^ Tregs with a CD44^hi^ CD62L^lo^ eTreg phenotype (*n* = 13 mice per group). Rel. MFI = relative MFI normalized to CD25 WT controls. Each symbol represents an individual mouse (**b**, **c**; mean ± SD). NS, not significant (*p* > 0.05), **p < 0.01, ***p < 0.001, ****p < 0.0001 (two-tailed Student’s *t* test). Data are representative of, or pooled from (**b**, **c**) three independent experiments

Previous work has suggested that IL-2 primarily controls the homeostasis of resting CD62L^hi^ CCR7^hi^ cTregs^27^. Thus, the increased expression of mRNAs and associated pathways in CD25 KO peripheral Tregs might reflect a shift in Treg subsets rather than a normal requirement for IL-2R-dependent gene repression. The RNA-seq data of individual genes support this view, since expression of cTreg mRNAs (*Sell*, *Ccr7*, *Ly6c1*, and *Bcl2*) was reduced in CD25-deficient peripheral Tregs, while eTreg mRNAs, including well-known Treg functional molecules (*Cd44*, *Icos*, *Klrg1*, *Mki67*, *Tigit*, *Irf4*, *Prdm1, Il10*, *Gzmb*, and *Fgl2*), were overexpressed (Fig. 8a).

Consistent with the RNA-seq data, flow cytometry revealed increased expression of the eTreg markers TIGIT, Klrg1, and ICOS among peripheral CD25-deficient Tregs (Fig. 8b). In addition, these CD25 KO Tregs contained a higher fraction of CD44^hi^ CD62L^lo^ eTregs (Fig. 8c). However, expression of Ly-6C, a marker of resting cTregs, was minimally reduced even though *Ly6c1* mRNA was downregulated ~2.5-fold in CD25 KO Tregs. Thus, some of the changes detected in peripheral CD25 KO Tregs by RNA-seq appear to reflect a shift from cTregs to eTregs, rather than loss of IL-2R-dependent gene repression.

YFP^+^ Tregs from CD25^flox/Foxp3eGFP-Cre-ERT2/R26Y^ mice were FACS-purified and stained for Foxp3, Ki67, and Bcl-2 by intracellular flow cytometry. Foxp3 expression among peripheral CD25 KO Tregs was reduced by ~30% at the mRNA and protein levels, while Bcl-2 mRNA and protein expression were reduced by ~50%. Ki67 expression was increased twofold at the mRNA level, while protein expression was indistinguishable between CD25 KO and WT Tregs (Fig. 8a, b). Collectively, these results suggest that abrogation of IL-2R signaling impairs survival of Tregs, particularly cTregs, while leaving proliferative function intact.

### Increased apoptosis among peripheral CD25 KO Tregs

**Fig. 9:**
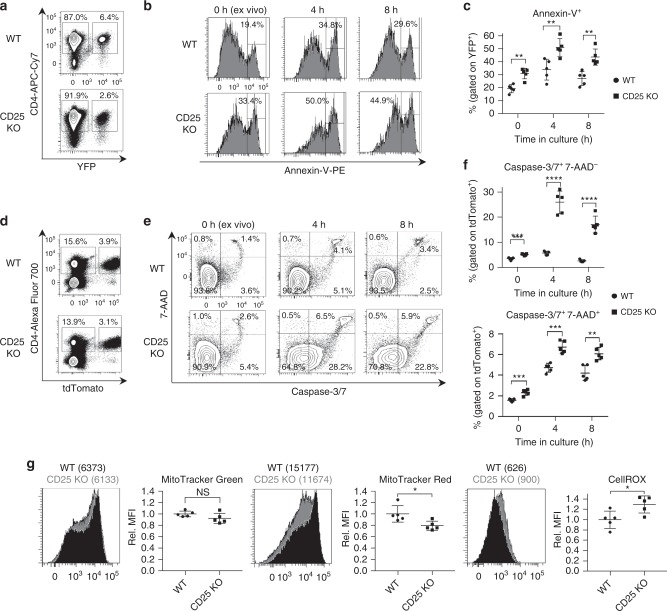
Increased apoptosis and mitochondrial dysfunction in CD25-deficient peripheral Tregs. **a** Representative contour plots and gating strategy for YFP^+^ Tregs among splenic CD4^+^ T cells bead-purified from CD25^flox/Foxp3eGFP-Cre-ERT2/R26Y^ mice (shown as CD25 KO) and reporter controls 10–14 days after tamoxifen induction. **b** Representative histograms and (**c**) scatter plot showing Annexin-V staining, performed ex vivo and after 4 h and 8 h incubation in culture media alone (n = 5 mice per group). Cells are gated on the YFP^+^ population shown in (**a**). **d** Representative contour plots and gating strategy for tdTomato^+^ Tregs among splenic CD4^+^ T cells from CD25^flox/Foxp3eGFP-Cre-ERT2/R26TD^ mice (shown as CD25 KO) and reporter controls evaluated 10–14 days after tamoxifen induction. **e** Representative contour plots and (**f**) scatter plots showing staining with caspase-3/7 enzyme substrate and 7-AAD (*n* = 5 mice per group). Cells are gated on the tdTomato^+^ population shown in (**d**). **g** Representative histograms and scatter plots of MFI data for tdTomato^+^ cells stained with MitoTracker Green, MitoTracker Red, and CellROX oxidative stress detection reagent (*n* = 5 mice per group). Rel. MFI = relative MFI normalized to CD25 WT controls. Each symbol represents an individual mouse (mean ± SD). NS, not significant (*p* > 0.05), *p < 0.05, **p < 0.01, ***p < 0.001, ****p < 0.0001 (two-tailed Student’s *t* test). Data are representative of (**a**, **b**, **d**, **e**, **g**) or pooled from (**c**, **f**, **g**) two independent experiments

To directly quantify cell survival in peripheral CD25 KO Tregs, Annexin-V staining was performed in splenocytes from CD25^flox/Foxp3eGFP-Cre-ERT2/R26Y^ mice and reporter controls 10–14 days post induction. The percentage of Annexin-V^+^ events was greater among CD25 KO Tregs stained ex vivo, and after resting in culture for 4 and 8 h (Fig. 9a–c). To obtain direct measurements of apoptosis, pro-apoptotic caspase-3/7 enzyme activity was assayed. When measured in CD25^flox/Foxp3eGFP-Cre-ERT2/R26TD^ mice and reporter controls, the proportion of caspase-3/7^+^ apoptotic cells, as well as 7AAD^+^ dead cells, was significantly increased among CD25 KO Tregs across the same time points (Fig. 9d–f).

### CD25 KO Tregs show signs of impaired metabolic homeostasis

Mitochondrial dysfunction among peripheral CD25 KO Tregs represents a likely mechanism underlying both the impaired lipid homeostasis suggested by IPA results (Fig. 6f) and the increased vulnerability of these cells to apoptosis. Accordingly, assessments of mitochondrial content, mitochondrial membrane potential, and cellular oxidative stress were performed using the fluorogenic dyes MitoTracker Green FM, MitoTracker Red FM, and CellROX Deep Red reagent, respectively. MitoTracker Green FM accumulates in the mitochondria regardless of mitochondrial membrane potential. Comparable MitoTracker Green FM staining observed in peripheral CD25 KO and WT Tregs indicates that mitochondrial content is unchanged. However, MitoTracker Red FM staining in the CD25 KO group was significantly diminished when compared with WT. Since accumulation of MitoTracker Red FM in the mitochondria is potential dependent, this result suggests that CD25 deficiency leads to a reduction in mitochondrial membrane potential. Furthermore, reactive oxygen species (ROS), which are likely to originate from the mitochondria and trigger apoptotic pathways^41,42^, were increased in CD25 KO Tregs (Fig. 9g). Notably, peripheral CD25 KO Tregs exhibited a host of transcriptional changes, such as increased levels of pro-apoptotic caspases, *Apaf1*, *Bak1*, and *Bid*, and decreased levels of *Bcl2* and *Bcl2l1*, consistent with the experimentally observed reduction in cell viability, as well as impaired expression of numerous genes involved in the biosynthesis of cholesterol and other lipids (Table 1). Thus, increased apoptosis of CD25 KO Tregs is accompanied by disruption of lipid homeostasis leading to mitochondrial dysfunction and oxidative stress.

**Table 1:** CD25-deficient Tregs show differential mRNA expression related to apoptosis and metabolism

Gene	Fold change (CD25 KO > WT)
*Casp3*	1.9
*Casp1*	1.7
*Casp4*	1.7
*Gas2*	1.5
*Apaf1*	1.3
*Bak1*	1.2
*Bid*	1.2
*Casp7*	1.2
**Gene**	**Fold change (WT > CD25 KO)**
*Ldlr*	2.6
*Bcl2*	2.0
*Ksr2*	1.9
*Dhcr24*	1.8
*Mvd*	1.8
*Lss*	1.7
*Fdps*	1.6
*Idi1*	1.6
*Mvk*	1.6
*Cyp51*	1.5
*Bcl2l1*	1.2
*Mcl1*	1.0

Selected genes related to apoptosis or lipid/cholesterol biosynthesis, evaluated by RNA-seq in splenic CD25 KO Tregs from tamoxifen-induced CD25^flox/Foxp3eGFP-Cre-ERT2/R26Y^ mice. Statistical significance is FDR < 0.01 for all comparisons (with the exception of *Mcl1*, where no difference was observed)

## Discussion

Past results have demonstrated that IL-2R signaling is required for functional programming of Tregs^7^. Our findings using CD25^cKO^ mice indicate that Tregs that do not receive an IL-2 signal are devoid of functional activity, as autoimmunity associated with this model is essentially identical to Foxp3-deficient scurfy mice, which lack Tregs altogether^8^. Moreover, autoimmunity was prevented in CD25^cKO^ and CD25^gKO^ mice after adoptive transfer of splenocytes, where essentially the only donor cells that persistently engrafted were Tregs. This finding is analogous to past results from our laboratory where purified Tregs readily prevented autoimmunity in IL-2Rβ-deficient mice^36^, further supporting the conclusion that Tregs are not functional in the absence of IL-2R signaling. Our study also clarifies that the slower tempo of lethal autoimmunity associated with germline inactivation of *Il2ra*^43^, *Il2rb*^44^, or *Il2*^45^ when compared with CD25^cKO^ mice is because IL-2R signaling is absent in both Tregs and autoreactive T cells in the former mice. Thus, impaired IL-2R signaling in autoreactive T cells accounts for this overall slower disease progression when compared with scurfy and CD25^cKO^ mice that harbor wild-type autoreactive T cells.

The complete inability of Tregs from CD25^cKO^ mice to suppress autoreactive T cells is consistent with an essential IL-2R-dependent step for functional programming of Tregs in the thymus. If thymic Tregs in CD25^cKO^ mice possessed significant functional competence, these Tregs should have exhibited some capacity to suppress autoreactive T cells in the periphery, but this was not observed. Moreover, phenotypic and gene profiling of thymic Tregs revealed substantial transcriptional changes among Tregs from CD25^cKO^ mice, including consistently lower expression of most Treg functional molecules. Given the role of STAT5 in regulating Foxp3 expression^2,7,37^, the nearly normal levels of Foxp3 protein in thymic Tregs from CD25^cKO^ mice were somewhat surprising, and suggest that IL-2 regulates one or more essential, Foxp3-independent targets during Treg development. The molecular basis for IL-2R-dependent Treg maturation remains to be determined.

By necessity, deletion of CD25 by Foxp3-Cre acts later in the Treg developmental pathway than germline deletion of CD25. Some aspects of the thymic phenotype associated with CD25^gKO^ mice, i.e., lower abundance of pSTAT5, Foxp3, CD39, CD73, Ki67, and Bcl-2, were more severe than found for CD25^cKO^ mice, suggesting a defect that occurs upstream of Foxp3-Cre-mediated ablation of CD25. These data support a two-stage model of IL-2R-dependent thymic Treg development. An early IL-2R-dependent step, preserved in CD25^cKO^ but not CD25^gKO^ mice, may potentiate initial upregulation of Foxp3 as well as survival and proliferation of thymic Treg precursors. The second step, associated with CD25^cKO^ mice, would then further potentiate Foxp3 expression and other essential aspects of Treg functional maturation. Nevertheless, the absence of this second step alone is sufficient to fully abolish Treg function^7^. It is noteworthy that ex vivo pSTAT5 was significantly reduced, but not abrogated, in developing Tregs from CD25^cKO^ and CD25^gKO^ mice. This result, as well as past findings that Foxp3 expression was lower in Tregs from IL-2Rβ-deficient mice^46,47^ when compared with CD25^cKO^ or CD25^gKO^ mice, implies that IL-7, IL-15, or other cytokines may act redundantly with IL-2 in promoting initial development of thymic Tregs. Nevertheless, our data clarify that other γc-dependent cytokines cannot compensate for a lack of IL-2R signaling in thymic Treg maturation.

Besides an indispensable role during thymic development, IL-2 also provides essential signaling for Tregs in the periphery. Although TCR^17–20^, co-stimulatory^21,22^, TNFRSF^23,24^, and IL-33^25,26^ signaling support the activation and proliferation of peripheral Tregs, fate mapping of this population after selective CD25 deletion indicates that all mature Tregs in lymphoid and non-lymphoid tissues require IL-2 for long-term persistence. Residual Tregs examined early after inducible CD25 deletion were skewed toward an eTreg phenotype, confirming prior reports that cTregs are particularly IL-2 dependent^27,28^. The absence of autoimmunity in this setting implies that the increase in eTregs is a direct physiological consequence of IL-2 deprivation. This differs from our assessment of CD4^+^ Foxp3^+^ T cells from the thymus and periphery of autoimmune and “cured” CD25^cKO^ and CD25^gKO^ mice, where skewing toward an eTreg phenotype arises as a consequence of inflammation associated with systemic autoimmunity.

The fundamental deficiency associated with CD25 KO peripheral Tregs lies with survival rather than proliferation, as these cells exhibited normal protein expression of the proliferative marker Ki67, but reduced expression of Bcl-2 mRNA and protein. Increased apoptosis among CD25 KO Tregs appears to be a result of dysregulated cellular metabolism, as evidenced by transcriptional downregulation of key enzymes involved in cholesterol and lipid biosynthesis as well as the crucial metabolic regulator kinase suppressor of Ras 2 (*Ksr2*). Recent evidence has established that regulating the homeostatic balance between glycolysis-driven fatty acid synthesis and fatty acid oxidation (FAO) has major implications for Treg maintenance and function^48,49^. Our study corroborates previous findings that lipid metabolism is integral to metabolic fitness, survival, and suppressive capacity among peripheral Tregs^50^, and highlights a critical role for IL-2 in this non-conventional regulatory process. Future studies will more directly assess the role of IL-2R signaling in Treg metabolism.

A previous report found that anti-apoptotic Mcl-1^51^, rather than Bcl-2, is essential for Treg survival and implicated Mcl-1 in IL-2-dependent homeostasis of Tregs. However, Treg-specific deletion of Mcl-1 produced lethal autoimmunity as early as 4 weeks of age, a much shorter time frame than the potential role we detected for Bcl-2 in supporting IL-2-dependent Treg survival. Moreover, the conclusion that Treg survival is Bcl-2-independent was based on competitive bone marrow transplantation, which more likely assesses homeostatic expansion rather than survival. Notably, our RNA-seq data revealed abundant expression of *Mcl1* that was comparable between splenic CD25 KO Tregs and controls. We therefore favor the notion that Mcl-1 regulates Treg survival independently of IL-2R signaling, while suggesting that Bcl-2 contributes to physiological IL-2-dependent Treg homeostasis.

Several studies have suggested that IL-2 is required to maintain Treg identity^2,3,7,15^. However, the relatively modest reduction in Foxp3 expression among peripheral CD25 KO Tregs and absence of a well-defined “ex-Treg” population after CD25 deletion argue against this. Neither peripheral lymphoid tissues nor the small intestinal lamina propria, a site known for its abundance of peripherally induced Tregs with greater phenotypic plasticity than their thymic-derived counterparts^30,52^, contained “ex-Tregs” in substantial numbers. Although we cannot directly discriminate thymic- and peripherally derived Tregs, our findings indicate that both remain stable in the absence of IL-2R signaling. At most, after prolonged absence of IL-2R signaling, Tregs may transiently become “ex-Tregs” before undergoing apoptosis, a mechanism which could account for the detection of several effector cytokine mRNAs in CD25-deficient peripheral Tregs.

Patterns of IL-2R-dependent gene regulation were highly distinctive in the thymus, where IL-2R signaling seems to predominantly drive gene repression, and the periphery where it primarily supports gene activation. Nevertheless, modest overlap was observed in IL-2R-dependent gene activation in thymic and splenic Tregs, particularly for regulatory activities related to survival, immune function, and energy homeostasis. By sustaining these fundamental pathways to support viability and expansion of Treg precursors in the thymus, IL-2R signaling appears to serve as a necessary prerequisite for generating Tregs with the potential for immunosuppression. However, an obligate IL-2 requirement for Treg survival is maintained after emigration to the periphery. Thus, some effects of IL-2R signaling on Tregs depend upon cell context, while other outcomes are common irrespective of anatomical location. With respect to this latter point, IL-2R-dependent gene regulation has also shown overlapping activities in Treg and T effector cells^53^. One caveat to this conclusion is that in the absence of IL-2R signaling, cTregs predominate in the thymus while eTregs dominate the periphery, and some IL-2R-dependent transcriptional differences may be secondary to this altered subset distribution. Notwithstanding this limitation, the dual action of IL-2R signaling has several precedents. For example, IL-7 and IL-15 also serve dual roles in the immune system, supporting development of T cells and NK cells, respectively, from less committed precursors only to later contribute to the homeostasis of naive T and memory T cells^54–56^. Thus, we favor the view that thymic Treg maturation depends on a unique IL-2R-dependent gene program distinct from IL-2R-dependent activities that regulate peripheral Treg homeostasis.

## Methods

### Study design

In this study, we aimed to determine the molecular mechanisms through which IL-2 signaling governs the development and survival of Tregs, by evaluating the phenotypes of mice with germline or Treg-targeted mutations in the *Il2ra* gene (CD25). In particular, the use of a tamoxifen-inducible CD25 knockout model allowed the impact of IL-2 on peripheral Tregs to be separated from IL-2-mediated effects within the thymus. In vitro and in vivo mouse studies were not randomized or blinded. CD25 WT littermates were used as experimental controls for all knockout strains. Identities of mouse strains were blinded for scoring of histological inflammation in H&E tissue sections. We chose sample sizes based on estimates from initial results, as well as previously published phenotypic data from other IL-2 signaling pathway mutations, in order to ensure appropriate power.

### Mice

Animal studies were reviewed and approved by the Institutional Animal Care and Use Committee at the University of Miami. *Il2ra*^−/−^ (CD25^gKO^), Foxp3^eGFP-Cre-ERT2^, Foxp3^YFP/Cre^, R26^flox/stop/YFP^, R26^flox/stop/tdTomato^, R26^FLPe^, CD45.1 congenic, and CD90.1 congenic mice, all on the C57BL/6 genetic background, were obtained from Jackson Laboratory. Foxp3/RFP mice (kindly provided by R.A. Flavell) were described previously^57^. Sperm from CD25^flox/flox^ mice (*Il2ra*^tm1a(EUCOMM)Wtsi^; EMMA ID: EM 08466) was first obtained from the Knockout Mouse Project (KOMP) Repository (https://www.komp.org). Mice with this allele were generated by in vitro fertilization using C57BL/6 donor oocytes, and resulting males were crossed with FLP recombinase-expressing transgenic females in order to eliminate *lacZ* and *neo* reporter constructs. All strains were bred within the specific pathogen-free animal facility at the University of Miami. Genotyping of strains was performed by PCR amplification using the primers and protocols recommended by Jackson Laboratory. For CD25^flox/Foxp3-YFP/Cre^ (CD25^cKO^) mice, genotyping was performed with custom primers (forward primer: 5′-ATCTCTGCGGCCAAAGTTCA-3′, reverse primer: 5′-TGCTGCAAAGACCCTGAGAC-3′) run for 35 cycles with an annealing temperature of 58 °C (Supplementary Fig. 1).

CD25^flox/Foxp3eGFP-Cre-ERT2/R26Y^ mice, CD25^flox/Foxp3eGFP-Cre-ERT2/R26TD^ mice, and respective CD25 WT reporter controls were treated with tamoxifen by intraperitoneal injection. Tamoxifen was dissolved in corn oil and injected daily for 5 consecutive days at a dose of 70 mg/kg.

For “curing” CD25^cKO^ and CD25^gKO^ mice, 1.0–1.5 × 10^7^ unfractionated WT splenocytes were adoptively transferred into 1–2 -day-old neonates by injection into the superficial facial vein. Congenic CD45.1 or CD90.1 C57BL/6 mice were used as donors in order to distinguish transferred cells from host-derived CD45.2^+^ or CD90.2^+^ populations (Supplementary Fig. 3).

### Histopathology

Tissues were obtained and processed as previously reported^58^, with major organs collected at necropsy, fixed in 10% neutral buffered formalin, sectioned, and stained with H&E. The tissues were coded and examined by a board certified veterinary pathologist. Inflammation was scored as: mild (+ , < 10%), moderate (+ + , 10–40%), and severe (+ + + , > 40%).

### Cell preparation and purification

Single-cell suspensions from the thymus, spleen, mesenteric lymph node, and Peyer’s patches were prepared by mechanical disruption. Intraepithelial and lamina propria lymphocytes (IEL and LP, respectively) were prepared by dissecting the small intestine from 0.5 cm below the pyloric sphincter to 1 cm above the cecum, and flushing the lumen with wash buffer consisting of Ca^2+^- and Mg^2+^-free HBSS containing 2.5% newborn calf serum (NCS), 100 U/mL penicillin, 100 µg/mL streptomycin, and 2 mM L-glutamine. After removing Peyer’s patches, intestines were cut into pieces of <3 mm and washed to remove residual mucus. For LP isolation, intestinal pieces were resuspended in Ca^2+^- and Mg^2+^-free HBSS containing 10% fetal bovine serum (FBS), 5 mM EDTA, and 15 mM HEPES, and incubated twice for 15 min at 37 °С with gentle agitation (15 rpm) to isolate IELs. Intestinal pieces were then minced finely with scissors and digested by incubating with wash buffer containing 300 U/mL collagenase type 3 and 10 µg/mL deoxyribonuclease I (Worthington Biochemical) for 1 h at 37 ^o^C. LP lymphocytes and IELs were purified on a 40/70% Percoll (GE) gradient (centrifuging at 800*g* for 20 min at 20 °С).

To purify Tregs and Treg subsets by FACS, total CD4^+^ T cells were obtained using anti-CD4 magnetic MicroBeads (Miltenyi Biotec). The cells were then stained and sorted based on the expression of reporter dyes and cell surface markers using a BD FACS Aria-II system. Cells were usually >95% pure.

### RNA-seq

FACS-purified Tregs were resuspended in TRIzol (Thermo Fisher). Separation of the RNA phase was performed according to the manufacturer instructions. Purification of RNA was performed using the RNeasy Micro Kit (Qiagen), according to the manufacturer instructions. Quality control analysis, library generation, and RNA-seq were carried out by the Oncogenomics Core Facility at the University of Miami. Quality control analysis of RNA samples was performed using the Bioanalyzer 2100 platform (Agilent Technologies). Libraries were prepared using KAPA’s RNA Hyperprep protocol and sequenced on a 75 bp paired-end run using the Illumina NextSeq 500 High Output Kit (150 cycle; 400 M flow cell).

Reads from RNA-seq were mapped to the *Mus musculus* genome GRCm38 using STAR (ver.2.5.0) aligner^59^. Raw counts were generated based on Ensembl genes (GENCODE M13) with featureCounts (ver.1.5.0)^60^. DE genes between CD25 KO and WT mice were identified using DESeq2^61^, and determined by a threshold of false discovery rate (FDR) < 0.01. Pathway analysis was performed using QIAGEN’s IPA (QIAGEN). Significant pathways were selected with cutoffs of *p*-value < 0.05 and FDR < 0.1 for thymus and FDR < 0.01 for the spleen, and analyzed using EnrichmentMap^62^ for pathway overlapping network. Pathway network was visualized in Cytoscape (https://cytoscape.org).

### Antibodies and flow cytometry

Fluorochrome-labeled mAbs, biotin-labeled mAbs, and fluorochrome–streptavidin conjugates used in this study, with sources and staining concentrations, are listed in Supplementary Table 1. For surface staining, cells were initially incubated with 2.4G2 to block antibody binding to Fc receptors, washed with HBSS containing 3% FBS and 0.1% (w/v) sodium azide, incubated with antibodies for 20 min at 4 ^o^C, and washed again. For staining with biotinylated anti-CCR7 (4B12; eBioscience), incubation was performed at 37 ^o^C, a modification which was reported by the manufacturer to enhance labeling brightness and resolution. As previously reported^46^, cells were stained for phospho-STAT5 by fixation in paraformaldehyde (1.5% final concentration) at 37 °C for 10 min. After centrifugation, cells were permeabilized in 100% methanol, maintained on ice for 30 min, then washed twice in PBS containing 0.5% bovine serum albumin and 0.02% (w/v) sodium azide before incubation with fluorochrome-conjugated antibodies to phospho-STAT5 and selected surface and intracellular markers. Intracellular staining of Foxp3, Ki67, Bcl-2, CTLA4, and Helios was performed using the Foxp3/Transcription Factor Staining Buffer Set (eBioscience) according to the manufacturer’s instructions. For analysis of unfractionated splenocytes from tamoxifen-induced CD25^flox/Foxp3eGFP-Cre-ERT2/R26Y^ mice and reporter controls, intracellular Foxp3 staining was performed using the BD Cytofix/Cytoperm Fixation/Permeabilization Kit in order to preserve YFP fluorescent signal. In this procedure, samples were fixed according to the manufacturer’s instructions, then incubated at 4 ^o^C for 1 h in the presence of 0.4 µg eFluor 450 anti-Foxp3 (FKJ-16S), diluted in 50 µL of Perm/Wash buffer. Samples were run on BD LSRFortessa HTS or CytoFLEX (Beckman Coulter) flow cytometers, and analyzed using BD FACSDiva or FlowJO software. Overall, 500,000 events were typically collected for each sample.

### Cytokine production assay

Purified anti-CD3 and anti-CD28 (BD Pharmingen) were diluted to a concentration of 5 μg/mL in PBS and coated onto 96-well EIA/RIA plates. Unfractionated splenocytes were resuspended in complete media (CM), consisting of RPMI 1640 (VWR) supplemented with 5% FBS, 100 U/mL penicillin, 100 µg/mL streptomycin, 2 mM L-glutamine, and 0.05 mM β-mercaptoethanol. The cell suspension was applied to antibody-coated plates at a seeding density of 2 × 10^5^ cells per well. Cultures were harvested after incubating at 37 ^o^C for 24 h. For each sample, culture supernatants were pooled from three replicate wells. Cytokine levels in supernatants were assayed by flow cytometry using the BD Cytometric Bead Array (CBA) Mouse Th1/Th2/Th17 Cytokine Kit according to the manufacturer’s instructions. Samples were run on a BD LSRFortessa HTS flow cytometer and analyzed using BD FACSDiva software.

### Apoptosis, mitochondrial, and oxidative stress assays

For Annexin-V staining, CD4-enriched splenocytes were first surface stained with APC-Cy7 anti-CD4 (GK1.5). Staining was performed using the PE Annexin-V Apoptosis Detection Kit (BD Pharmingen) according to the manufacturer’s instructions, with or without prior 37 ^o^C incubation in CM at a density of 2 × 10^6^/mL. Reagent volumes were scaled up to accommodate 3 × 10^5^ cells, in order to ensure an adequate number of labeled Tregs for analysis.

To measure caspase-3/7 activation, mitochondrial content, and mitochondrial membrane potential, unfractionated splenocytes were first surface stained with Alexa Fluor 700 anti-CD4 (RM4-5). For staining with MitoTracker (MT) Green FM and MitoTracker Red FM (Thermo Fisher), cells were immediately resuspended in CM at a density of 6 × 10^6^/mL. MT Green and MT Red, dissolved in DMSO, were added at a concentration of 150 µM, and cells were incubated for 15 min at 37 ^o^C. Cells were then washed once in HBSS containing 3% FBS and 0.1% (w/v) sodium azide, and resuspended in 1 mL. Cells were stained with SYTOX Blue Dead Cell Stain for flow cytometry (Thermo Fisher) according to the manufacturer’s instructions to exclude nonviable cells from analysis.

Staining with CellEvent Caspase-3/7 Green Flow Cytometry Assay Kit (Thermo Fisher) was performed with or without prior 37 ^o^C incubation in CM. In total, 6 × 10^6^ cells were resuspended in 1 mL of HBSS containing 3% FBS and 0.1% (w/v) sodium azide. CellEvent Caspase-3/7 Green Detection Reagent and SYTOX AADvanced Dead Cell Stain were added according to the manufacturer’s instructions, with a total incubation time of 30 min at room temperature.

For oxidative stress detection, unfractionated splenocytes were plated in CM at a density of 2 × 10^6^/mL. CellROX Deep Red Oxidative Stress Reagent, diluted in DMSO, was added to cells at a final concentration of 500 nM and incubated for 1 h at 37 ^o^C. Cells were then surface stained with FITC anti-CD4 (GK1.5), resuspended in 1 mL of HBSS with 3% FBS and 0.1% (w/v) sodium azide, and stained with SYTOX Blue Dead Cell Stain for flow cytometry (Thermo Fisher) according to the manufacturer’s instructions to exclude nonviable cells from analysis.

### Statistical analyses

Statistically significant differences between groups were evaluated using two-tailed Student’s *t* test, unless otherwise stated in individual figure legends. The following designations were used to convey test results: NS, not significant (*p* > 0.05), *p < 0.05, **p < 0.01, ***p < 0.001, ****p < 0.0001. Graphing and statistical testing were performed using GraphPad Prism Version 7.

### Reporting summary

Further information on experimental design is available in the Nature Research Reporting Summary linked to this article.

## Supplementary information

Supplementary Information

Reporting Summary

Source Data

## Data Availability

The RNA-seq data that support the findings of this study have been submitted to the National Center for Biotechnology Information/Gene Expression Omnibus database under accession number GSE121883. The source data underlying Figs. 1a, b, d, e, 2b, d–f, 3a, b, 4a–d, 5b, 7a–c, 8b, c, 9c, f, g, and Supplementary Figs 2b–e, 3c, e, f, and 4b are provided as a Source Data file. All other data that support the findings of this study are available from the corresponding author upon reasonable request.
