# Supplementary material for: Essential and non-overlapping IL-2Rα-dependent processes for thymic development and peripheral homeostasis of regulatory T cells
Source: Nat Commun. 2019 Mar 4;10:1037. doi: 10.1038/s41467-019-08960-1 (PMC6399264; doi:10.1038/s41467-019-08960-1)
Supplement: Supplementary file 1 — Supplementary Information [file 41467_2019_8960_MOESM1_ESM.pdf]

SUPPLEMENTARY INFORMATION

Toomer et al., Essential and non-overlapping IL-2Rα-dependent processes for thymic development and peripheral homeostasis of regulatory T cells

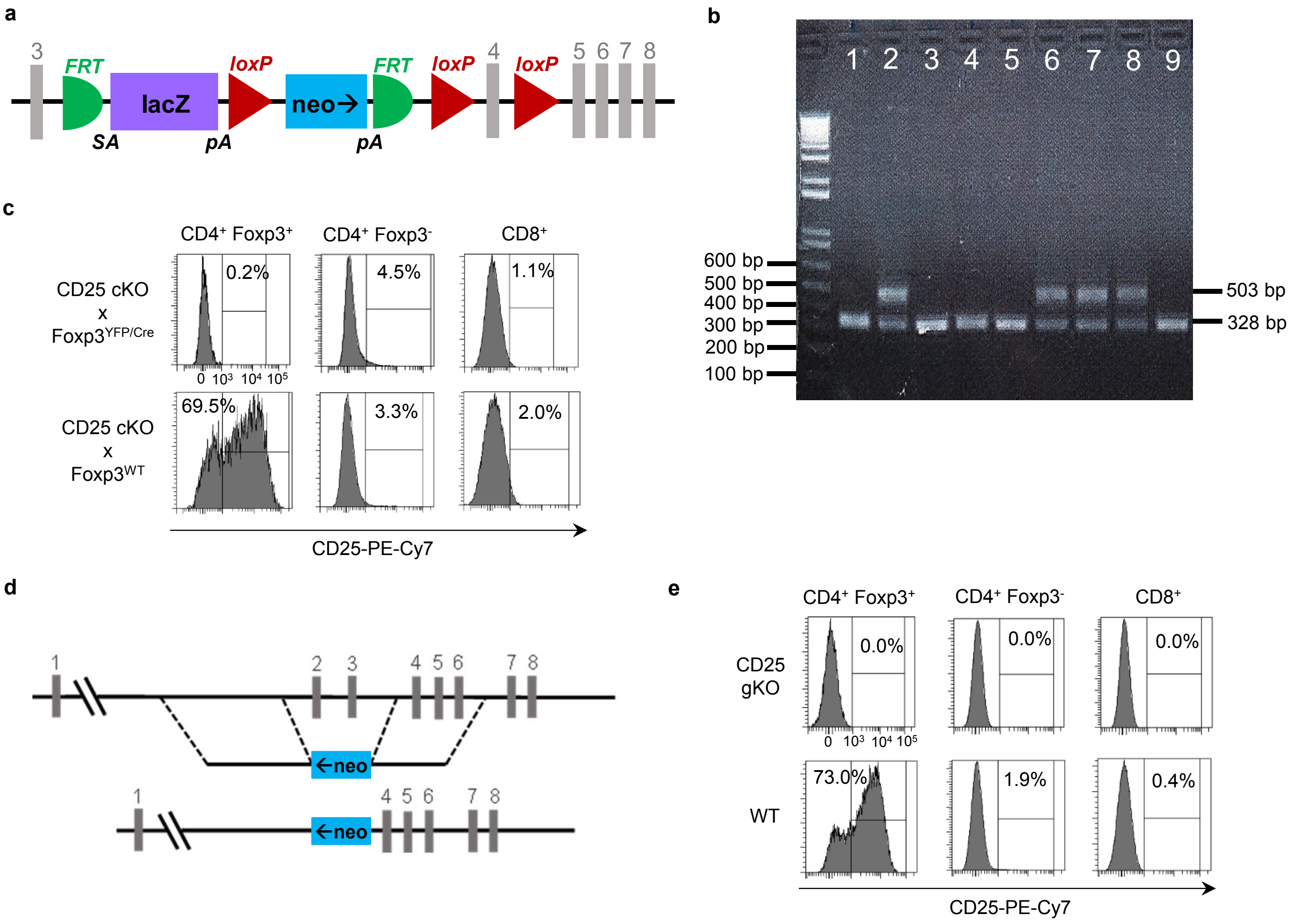

Supplementary Figure 1. Structure and validation of the *Il2ra* conditional KO and germline KO alleles. **a**, Diagram of the CD25$^{flox/Foxp3\text{-}YFP/Cre}$ (CD25^{cKO}) allele, with exons numbered. SA=splice acceptor site. pA=polyadenylation sequence. Adapted from http://www.mousephenotype.org (*Il2ra*$^{tm1a(EUCOMM)Wtsi}$). **b**, Validation of CD25^{cKO} allele by PCR. Male mice containing the CD25^{cKO} allele were crossed to FLP recombinase-expressing females to eliminate reporter constructs. Progeny of this cross (F1) (Lanes 1-8) and one WT control (Lane 9) were genotyped using primers targeted to the region between exons 3 and 4 of *Il2ra* (see Methods). In mice lacking the CD25^{cKO} allele, a single 328 bp fragment corresponding to the WT sequence of *Il2ra* is amplified (Lanes 1, 3, 4, 5, 9); in mice harboring the CD25^{cKO} allele, a second 503 bp fragment corresponds to the additional length of the vector insert after FLP-FRT recombination (Lanes 2, 6, 7, 8). Samples were run on 1.5% agarose gel containing 0.5 µg/mL ethidium bromide and visualized under UV light. **c**, Targeted abrogation of CD25 protein expression in CD4$^+$ Foxp3$^+$ T cells from the CD25^{cKO} model was confirmed by flow cytometry. Representative histograms, gated on the indicated T cell populations, show CD25 expression in CD25^{cKO} homozygous littermates with and without Foxp3$^{YFP/Cre}$. Mice were analyzed at 4 weeks of age. **d**, Structure of the CD25 germline KO (CD25^{gKO}) allele, showing the use of a targeting vector (middle) to eliminate exons 2 and 3 of *Il2ra* as previously described[1]. **e**, Abrogation of CD25 expression across T lymphocyte subsets in the CD25^{gKO} model was confirmed by flow cytometry. Representative histograms, gated on the indicated T cell populations, show CD25 expression in a CD25^{gKO} homozygote and WT littermate control. Mice were analyzed at 8 weeks of age.

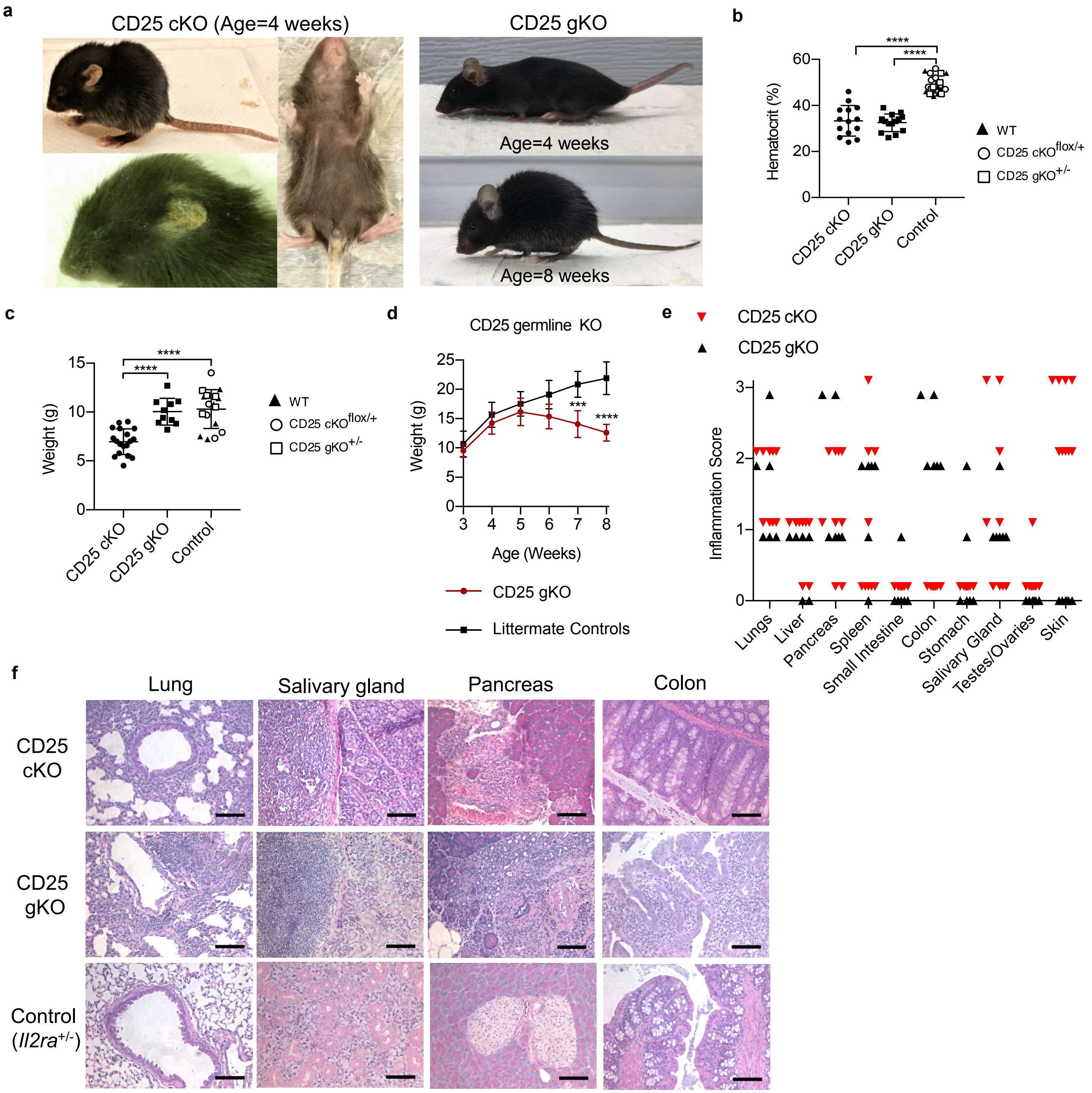

Supplementary Figure 2. Clinical and histological data from CD25^{cKO} and CD25^{gKO} mice.
a, Representative images of CD25$^{flox/Foxp3\text{-}YFP/Cre}$ (CD25^{cKO}) and CD25^{gKO} mice. **b**, CD25^{cKO} and CD25^{gKO} mice in the advanced stages of disease show significantly reduced hematocrit relative to WT and heterozygous littermate controls (CD25^{cKO} age=3-4 weeks; CD25^{gKO} age=7-8 weeks; n=6-14 mice per genotype). **c**, CD25^{cKO} mice, but not CD25^{gKO} mice, exhibit significant weight loss at 3-4 weeks of age when compared to WT and heterozygous littermate controls (n=5-18 mice per genotype). Each point represents an individual mouse (**b**,**c**; mean ± SD). **d**, Weight loss occurs gradually in the CD25^{gKO} model and is pronounced by 7-8 weeks of age (n=7-8 mice per time point; error bars mean ± SD). **e**, Scoring of histological inflammation in H&E stained tissue sections (1= Mild, <10%; 2=Moderate, 10-40%; 3=Severe, >40%) from CD25^{cKO} and CD25^{gKO} mice with established disease (CD25^{cKO} age=3-4 weeks; CD25^{gKO} age=7-8 weeks; n=6-9 mice per tissue and genotype). **f**, Representative H&E stained sections of lung, submandibular salivary gland, pancreas, and colon from CD25^{cKO} and CD25^{gKO} mice with established disease (CD25^{cKO} age=3-4 weeks; CD25^{gKO} age=7-8 weeks). 7-8 week old mice heterozygous for the CD25^{gKO} allele (*Il2ra*$^{+/-}$) were used as controls. Magnification 200X; scale bar 100 μm. ***P<0.001, ****P<0.0001 (two-tailed Student's *t* test).

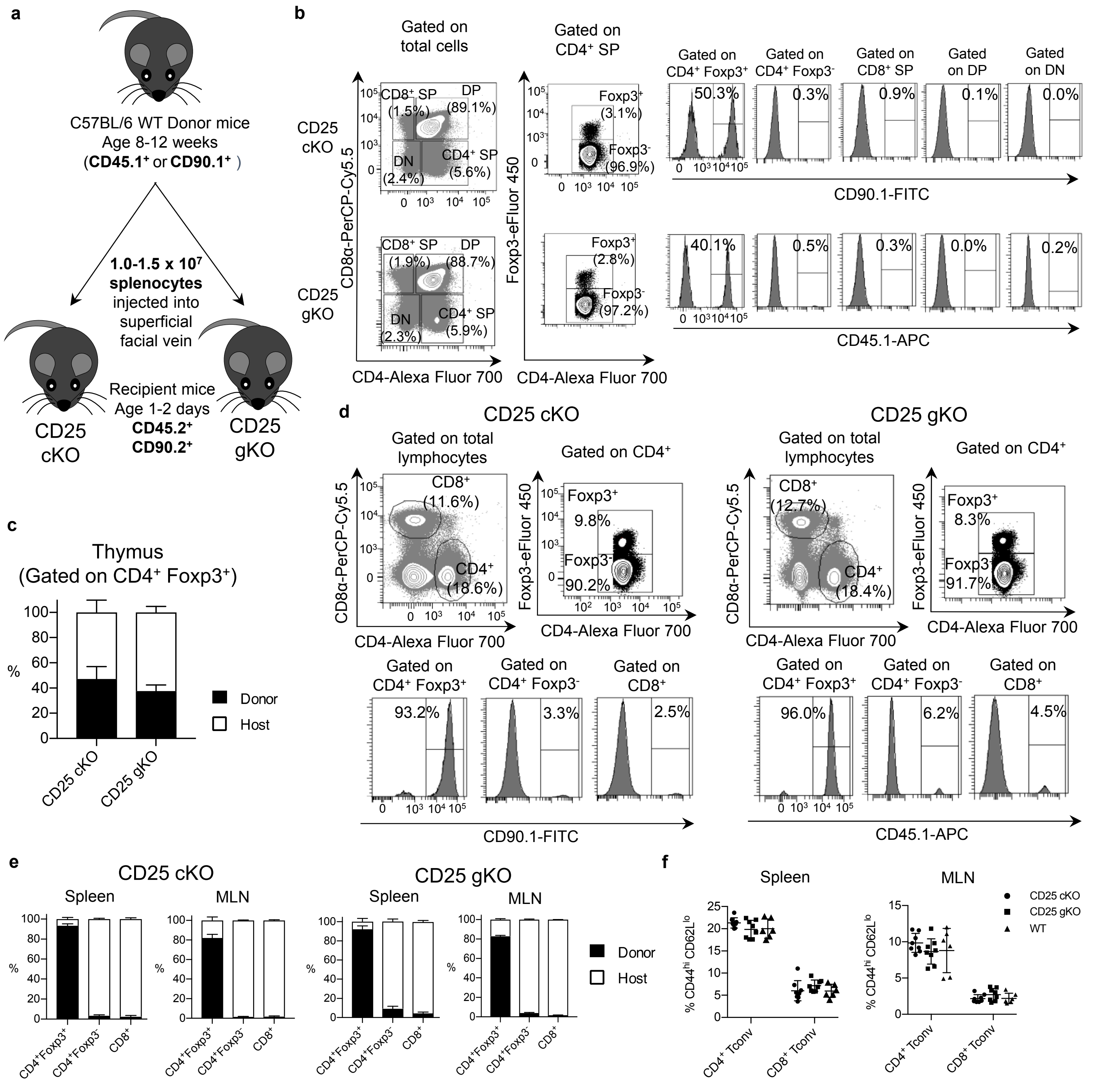

Supplementary Figure 3. Generation and characterization of 'cured' mice. a, CD25$^{flox/Foxp3\text{-}YFP/Cre}$ (CD25^{cKO}) and CD25^{gKO} mice were 'cured' of autoimmune disease by intravenous injection of 1.0-1.5 x 10^7 unfractionated WT splenocytes into neonates at 1-2 days of age. Engraftment of donor CD4$^+$ Foxp3$^+$ Tregs prevented development of autoimmunity, as 'cured' animals exhibited normal life spans and were clinically indistinguishable from WT. Donor T lymphocytes were distinguished from those of recipients by their expression of different allelic variants of CD45 or CD90, as shown. **b**, Representative contour plots and histograms with gating strategy to distinguish donor and host populations within the thymus of 'cured' mice. SP=single positive; DP=double positive; DN=double negative. **c**, Percentages of donor- and host-derived CD4$^+$ Foxp3$^+$ T cells in the thymus of 'cured' adult mice (age=7-8 weeks; n=8 mice per genotype). **d**, Representative contour plots and histograms with gating strategy to distinguish donor and host T cells in spleen of 'cured' adults. **e**, Percentages of donor- and host-derived T lymphocytes in the spleen and mesenteric lymph nodes (MLN) of 'cured' adult CD25^{cKO} and CD25^{gKO} mice (age=7-8 weeks; n=8 mice per genotype). **f**, Percentages of host-derived T conventional (Tconv) lymphocytes expressing a highly activated CD44^{hi} CD62L^{lo} phenotype in 'cured' CD25^{cKO} and CD25^{gKO} adults and WT controls (age=7-8 weeks; n=6-8 mice per genotype) (**c,e,f**; mean ± SD). Data are representative of (**b,d**) or pooled from (**c,e,f**) three independent experiments.

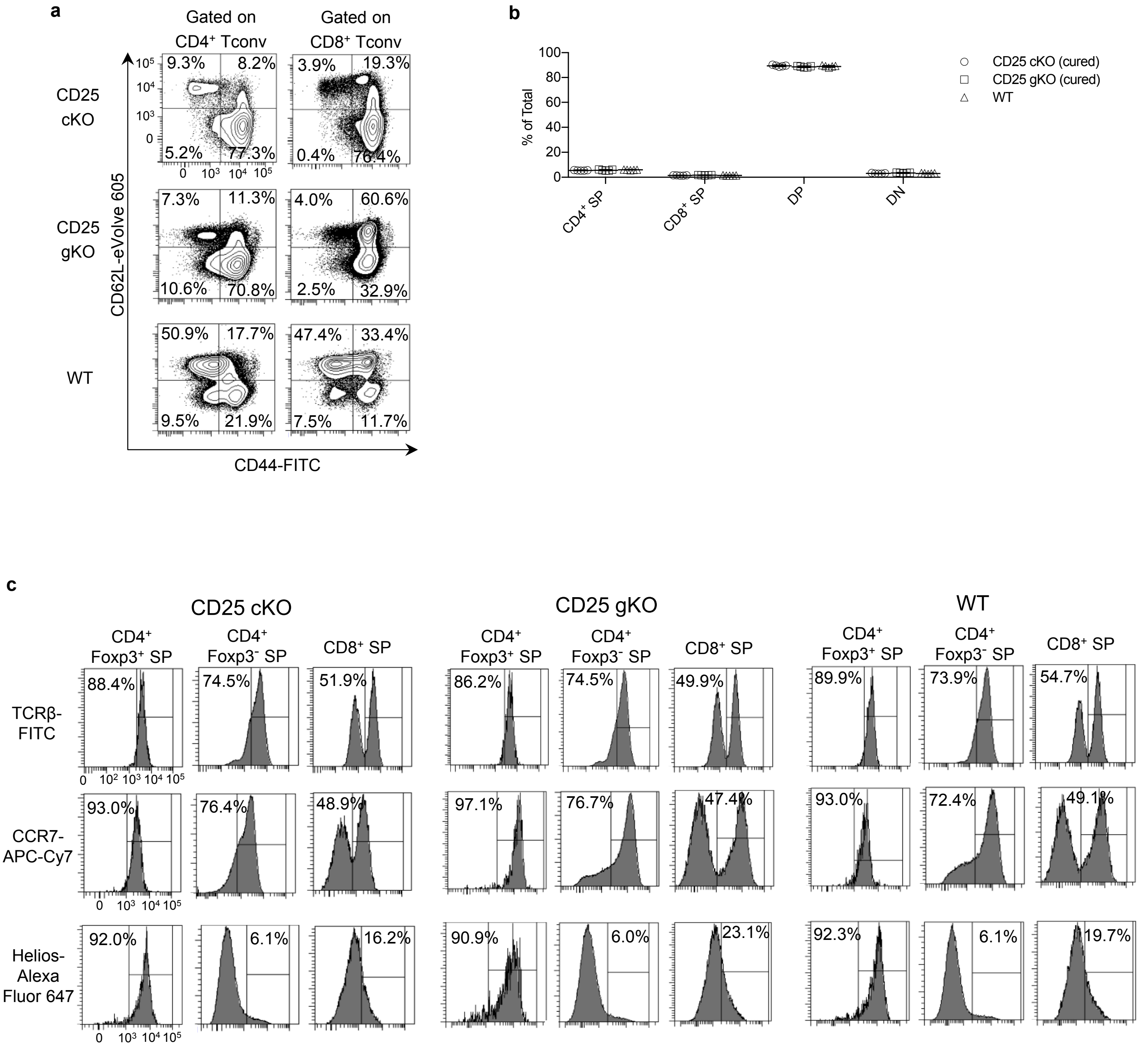

Supplementary Figure 4. Flow cytometry data from splenic Tconv cells and thymocytes in CD25^{cKO} and CD25^{gKO} mice. **a**, Representative contour plots showing CD44 and CD62L expression profiles and gating strategy among splenic CD4^{+} and CD8^{+} T conventional (Tconv) cells from CD25$^{flox/Foxp3\text{-}YFP/Cre}$ (CD25^{cKO}), CD25^{gKO}, and WT mice at 4 weeks of age. **b**, Scatter plot showing proportions of thymocytes belonging to the CD4^{+} and CD8^{+} single positive (SP), double positive (DP) and double negative (DN) subsets in 'cured' CD25^{cKO}, 'cured' CD25^{gKO}, and WT mice evaluated at 12-14 days of age (mean ± SD; n=5 mice per genotype). **c**, Representative histograms showing expression patterns and gating strategies for TCRβ, CCR7, and Helios in the indicated thymocyte subpopulations from 'cured' CD25^{cKO}, 'cured' CD25^{gKO}, and WT mice evaluated at 12-14 days of age. Data are representative of (**a,c**) or pooled from (**b**) three independent experiments.

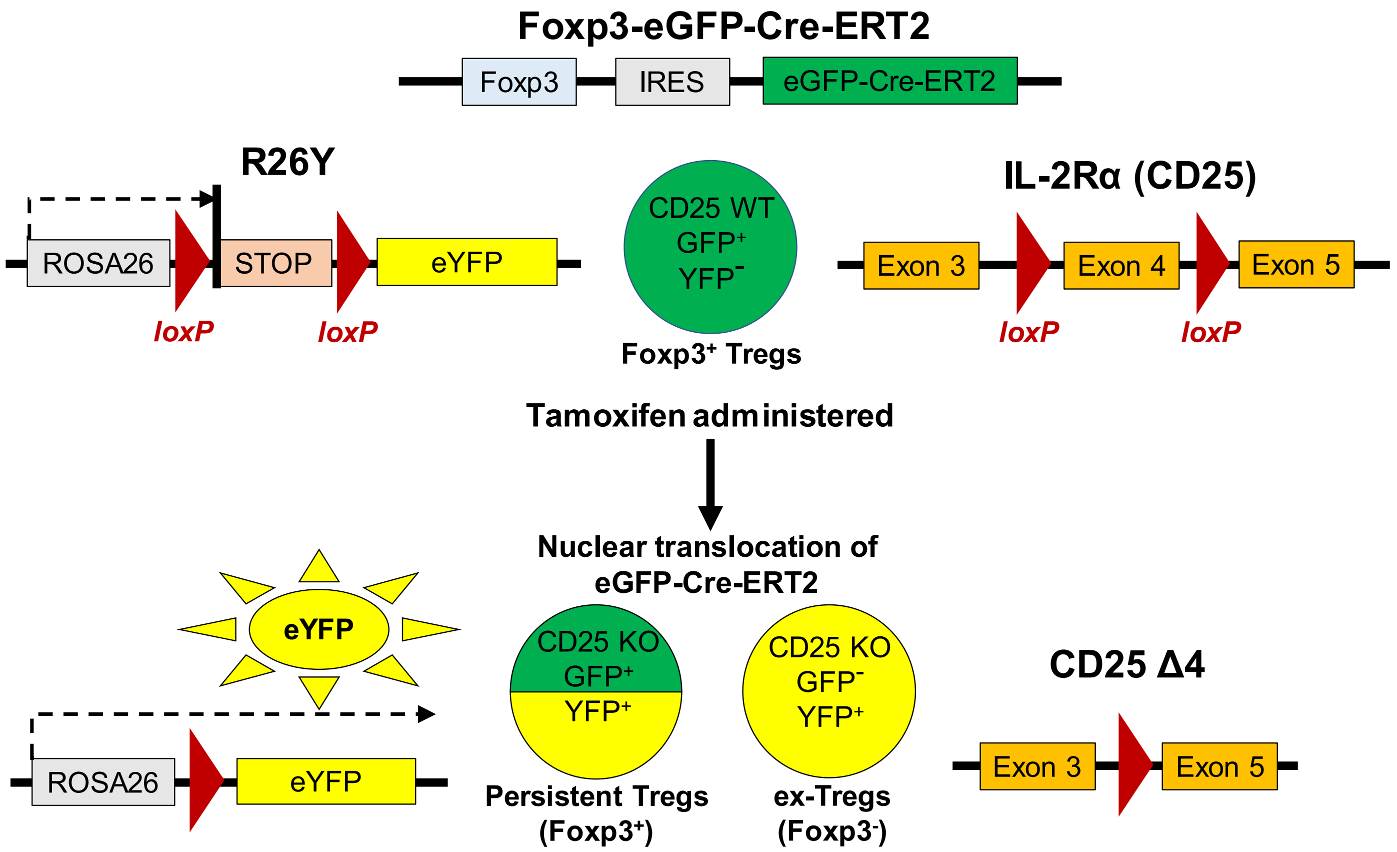

Supplementary Figure 5. Schematic of Treg fate mapping in the CD25$^{flox/Foxp3eGFP\text{-}Cre\text{-}ERT2/R26Y}$ mouse model. The CD25$^{flox/Foxp3eGFP\text{-}Cre\text{-}ERT2/R26TD}$ model uses the same system, with a tdTomato reporter replacing eYFP.

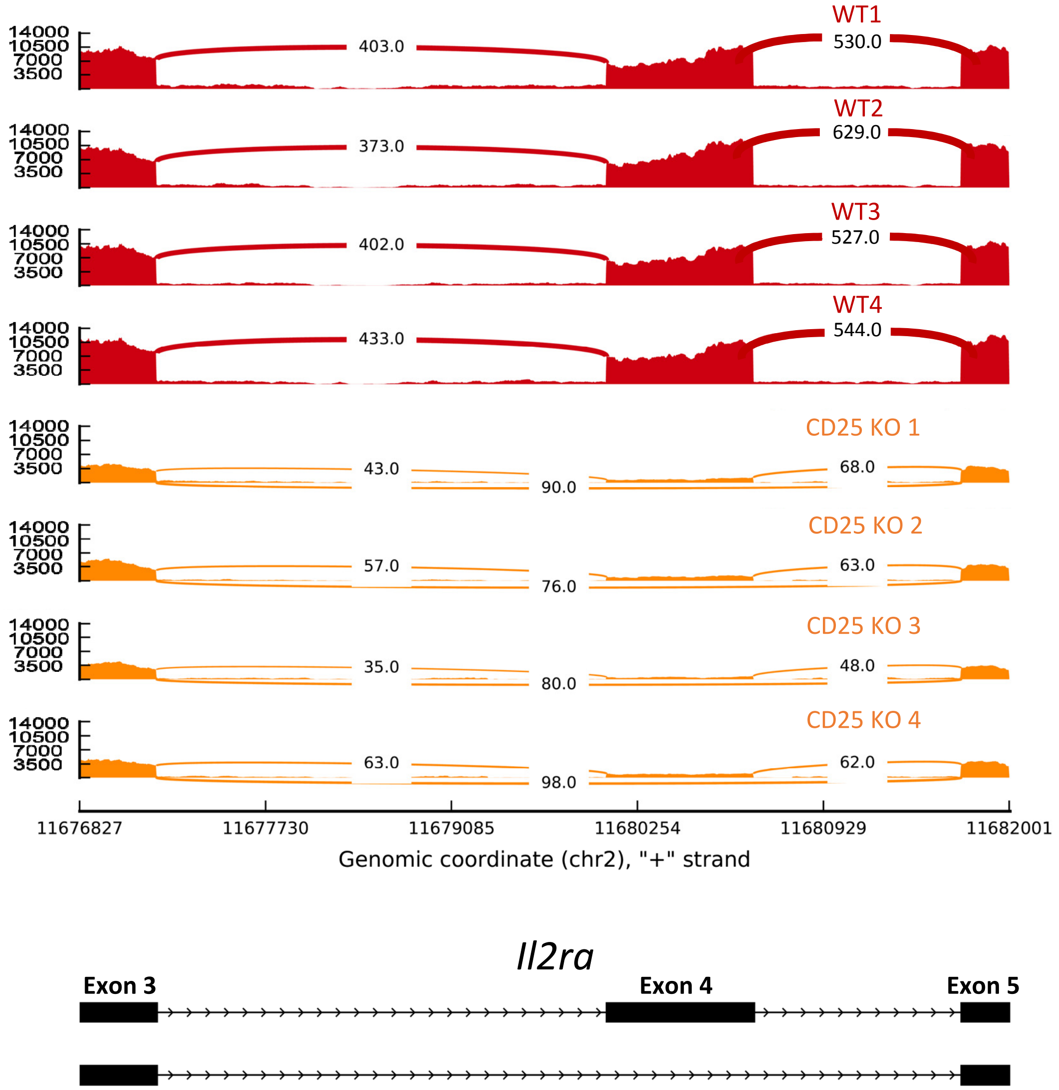

Supplementary Figure 6. Analysis of alternative splicing events in exon 4 of the *Il2ra* gene. RNA-seq data from YFP^{+} splenic Tregs in tamoxifen-induced CD25$^{flox/Foxp3eGFP-Cre-ERT2/R26Y}$ mice and WT reporter controls were analyzed using the rMATS program (http://rnaseq-mats.sourceforge.net/). Results from individual mice are expressed as Sashimi plots. Read densities are shown on the Y-axis, while junction-spanning read counts are shown on connecting arcs.

Thymus	Spleen
ACVR1	**ACVR1**
CCR3	**CCR3**
CCR4	**CCR4**
CCR8	**CCR8**
**CD274**	**CD274**
CD3E	**CD3E**
CD4	**CD4**
CD80	**CD80**
**CD86**	CD86
ICAM1	**ICAM1**
ICOS	**ICOS**
IFNGR1	**IFNGR1**
IL1RL1	**IL1RL1**
IL2RA	IL2RA
IL2RB	IL2RB
IL4RA	IL4RA
**JAK2**	**JAK2**
LTA	LTA
NFIL3	**NFIL3**
NOTCH2	NOTCH2
RUNX3	**RUNX3**
SOCS1	SOCS1
**STAT1**	**STAT1**
TNFRSF4	**TNFRSF4**
**ACVR2A**	ACVR1B
ICOSL	ACVR1C
IL10RA	**CCR5**
IL18R1	**CD28**
**IRF1**	**CD3D**
JUN	**CD3G**
NFATC2	**CD40LG**
PSEN1	**CXCR3**
PSEN2	**CXCR4**
SOCS3	**CXCR6**
STAT3	**GATA3**
TGFB1	**GRB2**
	**HAVCR2**
	IFNAR1
	**IFNG**
	IFNGR2
	**IL10**
	IL10RB
	**IL4**
	IL6RA
	JAG2
	**MAF**
	**NFATC1**
	**NFATC3**
	PIK3C2B
	**PIK3CA**
	PIK3CD
	PIK3R1
	PIK3R3
	PIK3R5
	**PTPN11**
	**STAT4**
	**TBX21**
	TGFBR1

WT>CD25 KO

CD25 KO>WT

Supplementary Figure 7. Differentially regulated genes within the Th1 and Th2 Activation Pathway identified by Ingenuity Pathway Analysis. Genes differentially regulated between WT and CD25 KO Tregs in both thymus and spleen are shown on gray background; genes differentially regulated in thymus or spleen exclusively are shown on white background. Genes downregulated in CD25 KO Tregs are shown in regular font, while those upregulated in CD25 KO Tregs are bolded.

mAb Target	Clone	mAb Label	Source	Staining Concentration	Catalogue Number
CD4	GK1.5	APC	BioLegend	4.0 μg/mL	100412
CD4	GK1.5	APC-Cy7	BioLegend	4.0 μg/mL	100414
CD4	GK1.5	FITC	Laboratory made	5.0 μg/mL	N/A
CD4	GK1.5	PE	BioLegend	2.0 μg/mL	100512
CD4	RM4-5	AlexaFluor700	Laboratory made	5.0 μg/mL	N/A
CD4	RM4-5	PE-Cy7	BD Pharmingen	0.5 μg/mL	552775
CD8α	53-6.7	APC	BD Pharmingen	4.0 μg/mL	553035
CD8α	53-6.7	APC-Cy7	BioLegend	0.5 μg/mL	100714
CD8α	53-6.7	Brilliant Violet 605	BioLegend	1:200 dilution	100743
CD8α	53-6.7	PerCP-Cy5.5	BioLegend	2.5 μg/mL	100734
CD25	PC61	APC	BioLegend	3.0 μg/mL	102012
CD25	PC61	PE	BioLegend	3.0 μg/mL	102008
CD25	PC61	PE-Cy7	BioLegend	1.25 μg/mL	102016
CD25	PC61	PE/Dazzle 594	BioLegend	2.0 μg/mL	102048
CD39	24DMS1	eFluor 660	eBioscience	2.0 μg/mL	50-0391-82
CD39	24DMS1	PE	eBioscience	2.0 μg/mL	12-0391-82
CD44	PGP-1	AlexaFluor 647	Laboratory made	5.0 μg/mL	N/A
CD44	PGP-1	FITC	Laboratory made	10 μg/mL	N/A
CD45.1	A20	APC	BioLegend	2.0 μg/mL	110714
CD45.1	A20	APC-Cy7	BioLegend	3.0 μg/mL	110716
CD45.2	104	APC	BioLegend	2.0 μg/mL	109814
CD45.2	104	APC-Cy7	BioLegend	3.0 μg/mL	109824
CD45.2	104	FITC	BioLegend	4.0 μg/mL	109806
CD62L	MEL-14	eVolve 605	eBioscience	1:100 dilution	83-0621-42
CD73	eBioTY/11.8	PE	eBioscience	0.5 μg/mL	12-0731-82
CD73	eBioTY/11.8	PE-Cy7	eBioscience	1.25 μg/mL	25-0731-82
CD90.1	HIS51	FITC	eBioscience	1.0 μg/mL	11-0900-85
CD90.1	HIS51	PerCP-Cy5.5	eBioscience	0.5 μg/mL	45-0900-82
Bcl-2	BCL/10C4	Alexa Fluor 647	BioLegend	4.0 μg/mL	633510
Bcl-2	BCL/10C4	PE	BioLegend	4.0 μg/mL	633508
CTLA4 (CD152)	UC10-4B9	PE	BioLegend	4.0 μg/mL	106306
CCR7 (CD197)	4B12	Biotin	eBioscience	5.0 μg/mL	12-1971-82
Foxp3	FKJ-16S	eFluor 450	eBioscience	2.0 μg/mL	48-5773-82
Helios	22F6	AlexaFluor 647	BioLegend	1:20 dilution	137218
ICOS (CD278)	15F9	PerCP-eFluor 710	eBioscience	2.0 μg/mL	46-9940-82
Ki67	B56	AlexaFluor 700	BD Pharmingen	1:50 dilution	561277
Klrg1	2F1/KLRG1	APC	BioLegend	0.5 μg/mL	138412
Klrg1	2F1	Biotin	eBioscience	1.0 μg/mL	13-5893-82
Klrg1	2F1	FITC	eBioscience	1.0 μg/mL	11-5893-82
Ly-6C	AL-21	APC-Cy7	BD Pharmingen	2.0 μg/mL	560596
Phospho-STAT5 (Y694)	47/Stat5[pY694]	Alexa Fluor 647	BD Phosflow	1:10 dilution	612599
TCRβ	H57-597	FITC	Laboratory made	10 μg/mL	N/A
TIGIT	GIGD7	PerCP-eFluor 710	eBioscience	2.5 μg/mL	46-9501-82

Streptavidin Conjugate Label	Source	Staining Concentration	Catalogue Number
APC	BioLegend	2.0 μg/mL	405207
APC-Cy7	BioLegend	2.5 μg/mL	405208
eFluor 450	eBioscience	2.0 μg/mL	48-4317-82
PE-Cy7	eBioscience	2.5 μg/mL	25-4317-82
PerCP-eFluor 710	eBioscience	2.0 μg/mL	46-4317-82
PE-CF594	BD Horizon	2.0 μg/mL	562284

Supplementary Table 1. Fluorochrome-labeled mAbs, biotin-labeled mAbs, and fluorochrome-streptavidin conjugates used in this study, with sources and staining concentrations. Laboratory made anti-CD4 (GK1.5) and anti-TCRβ (H57-597) were conjugated using fluorescein NHS Ester (Molecular Probes); laboratory made anti-CD4 (RM4-5) was conjugated using Alexa Fluor 700 NHS Ester (Molecular Probes); laboratory made anti-CD44 (PGP-1) was conjugated using fluorescein NHS Ester and Alexa Fluor 647 NHS Ester (Molecular Probes). Conjugation was performed according to the manufacturer’s instructions.

SUPPLEMENTARY REFERENCES

1. Willerford DM, Chen J, Ferry JA, Davidson L, Ma A, Alt FW. Interleukin-2 receptor α chain regulates the size and content of the peripheral lymphoid compartment. *Immunity* **3**, 521-530 (1995).
