# Supplementary material for: Essential and non-overlapping IL-2Rα-dependent processes for thymic development and peripheral homeostasis of regulatory T cells
Source: Nat Commun. 2019 Mar 4;10:1037. doi: 10.1038/s41467-019-08960-1 (PMC6399264; doi:10.1038/s41467-019-08960-1)
Supplement: Supplementary file 2 — Reporting Summary [file 41467_2019_8960_MOESM2_ESM.pdf]

nature research

Corresponding author(s): Thomas R. Malek

Reporting Summary

Nature Research wishes to improve the reproducibility of the work that we publish. This form provides structure for consistency and transparency in reporting. For further information on Nature Research policies, see Authors & Referees and the Editorial Policy Checklist.

Statistical parameters

When statistical analyses are reported, confirm that the following items are present in the relevant location (e.g. figure legend, table legend, main text, or Methods section).

n/a	Confirmed	
☐	☒	The exact sample size (n) for each experimental group/condition, given as a discrete number and unit of measurement
☐	☒	An indication of whether measurements were taken from distinct samples or whether the same sample was measured repeatedly
☐	☒	The statistical test(s) used AND whether they are one- or two-sided<br>*Only common tests should be described solely by name; describe more complex techniques in the Methods section.*
☐	☒	A description of all covariates tested
☐	☒	A description of any assumptions or corrections, such as tests of normality and adjustment for multiple comparisons
☐	☒	A full description of the statistics including central tendency (e.g. means) or other basic estimates (e.g. regression coefficient) AND variation (e.g. standard deviation) or associated estimates of uncertainty (e.g. confidence intervals)
☐	☒	For null hypothesis testing, the test statistic (e.g. F, t, r) with confidence intervals, effect sizes, degrees of freedom and P value noted<br>*Give P values as exact values whenever suitable.*
☒	☐	For Bayesian analysis, information on the choice of priors and Markov chain Monte Carlo settings
☒	☐	For hierarchical and complex designs, identification of the appropriate level for tests and full reporting of outcomes
☒	☐	Estimates of effect sizes (e.g. Cohen's d, Pearson's r), indicating how they were calculated
☐	☒	Clearly defined error bars<br>*State explicitly what error bars represent (e.g. SD, SE, CI)*

Our web collection on statistics for biologists may be useful.

Software and code

Policy information about availability of computer code

Data collection: *Provide a description of all commercial, open source and custom code used to collect the data in this study, specifying the version used OR state that no software was used.*

Data analysis: *Provide a description of all commercial, open source and custom code used to analyse the data in this study, specifying the version used OR state that no software was used.*

For manuscripts utilizing custom algorithms or software that are central to the research but not yet described in published literature, software must be made available to editors/reviewers upon request. We strongly encourage code deposition in a community repository (e.g. GitHub). See the Nature Research guidelines for submitting code & software for further information.

Data

Policy information about availability of data

All manuscripts must include a data availability statement. This statement should provide the following information, where applicable:

- Accession codes, unique identifiers, or web links for publicly available datasets
- A list of figures that have associated raw data
- A description of any restrictions on data availability

The RNA-seq data that support the findings of this study have been submitted to the National Center for Biotechnology Information/Gene Expression Omnibus

database (https://www.ncbi.nlm.nih.gov/geo) under accession number (to be available before publication). All other data that support the findings of this study are available from the corresponding author upon reasonable request.

Field-specific reporting

Please select the best fit for your research. If you are not sure, read the appropriate sections before making your selection.

☒ Life sciences ☐ Behavioural & social sciences ☐ Ecological, evolutionary & environmental sciences

For a reference copy of the document with all sections, see nature.com/authors/policies/ReportingSummary-flat.pdf

Life sciences study design

All studies must disclose on these points even when the disclosure is negative.

Sample size	We chose sample sizes based on estimates from initial results, as well as previously published phenotypic data from other IL-2 signaling pathway mutations, in order to ensure appropriate power.
Data exclusions	No data were excluded.
Replication	Typically 3 independent experiments were performed that contained multiple mice/group. One exception was FACS analysis of mitochondrial activity or size, where only 2 replicate experiments were performed.
Randomization	No randomization was used.
Blinding	Scoring of H & E slides was blinded. Other experiments were not.

Reporting for specific materials, systems and methods

Materials & experimental systems

n/a	Involved in the study	
☐	☒	Unique biological materials
☐	☒	Antibodies
☒	☐	Eukaryotic cell lines
☒	☐	Palaeontology
☐	☒	Animals and other organisms
☒	☐	Human research participants

Methods

n/a	Involved in the study	
☒	☐	ChIP-seq
☐	☒	Flow cytometry
☒	☐	MRI-based neuroimaging

Unique biological materials

Policy information about availability of materials

Obtaining unique materials	The CD25-flox/flox mice will be made available to qualified investigators after completion of an MTA. Other mouse strains are available from JAX laboratories.

Antibodies

Antibodies used	All antibodies are listed in the methods, with the clone names and fluorochromes used.
Validation	We used well-defined monoclonal antibodies to lymphocyte-associated molecules that exhibited the expected staining pattern on control cells.

Animals and other organisms

Policy information about studies involving animals; ARRIVE guidelines recommended for reporting animal research

Laboratory animals	All strains are listed in the methods. Both sexes were used, and we did not note any sex-related differences in our results. For CD25 germline knockout, and Foxp3-Cre-driven CD25 conditional knockout mice, animals were typically between 12 days and 8

weeks of age, as specified in individual figure legends. For tamoxifen-inducible CD25 knockout mice, experiments were initiated when animals were 7-10 weeks of age.

Wild animals	N/A
Field-collected samples	N/A

Flow Cytometry

Plots

Confirm that:

☒ The axis labels state the marker and fluorochrome used (e.g. CD4-FITC).

☒ The axis scales are clearly visible. Include numbers along axes only for bottom left plot of group (a 'group' is an analysis of identical markers).

☒ All plots are contour plots with outliers or pseudocolor plots.

☒ A numerical value for number of cells or percentage (with statistics) is provided.

Methodology

Sample preparation	Single cell lymphocyte suspensions from thymus, spleen, mesenteric lymph node, and Peyer's patches were prepared by mechanical disruption. Intraepithelial and lamina propria lymphocytes (IEL and LP, respectively) were prepared by dissecting the small intestine from 0.5 cm below the pyloric sphincter to 1 cm above the cecum, and flushing the lumen with wash buffer consisting of Ca2+- and Mg2+-free HBSS containing 2.5% newborn calf serum (NCS), 100 U/mL penicillin, 100 µg/mL streptomycin, and 2mM L-glutamine. After removing Peyer's patches, intestines were cut into pieces <3 mm and washed to remove residual mucus. For LP isolation, intestinal pieces were resuspended in Ca2+- and Mg2+-free HBSS containing 10% fetal bovine serum (FBS), 5 mM EDTA and 15 mM HEPES, and incubated twice for 15 minutes at 37° C with gentle agitation (15 rpm) to isolate IELs. Intestinal pieces were then minced finely with scissors and digested by incubating with wash buffer containing 300 U/mL Collagenase Type 3 and 10 µg/mL deoxyribonuclease I (Worthington Biochemical) for 1 hr at 37o C. LP lymphocytes and IELs were purified on a 40/70% Percoll (GE) gradient (centrifuging at 800 g for 20 minutes at 20° C).<br>To purify Tregs and Treg subsets by FACS, total CD4+ T cells were obtained using anti-CD4 magnetic MicroBeads (Miltenyi Biotec). The cells were then stained and sorted based on the expression of reporter dyes and cell surface markers.
Instrument	BD LSRFortessa HTS, BD FACS Aria-II, CytoFLEX S (Beckman-Coulter)
Software	BD FACSDiva Version 8.0, FlowJo Version 10.4.2
Cell population abundance	For FACS sorting, purity was determined by applying the same gating strategy used during sorting to post-sort fractions of 10,000-20,000 cells. Usually >95% of cells belonged to the population of interest. For FACS analysis, Typically 500,000 total non-gated events were collected per sample.
Gating strategy	Preliminary strategy consisted of gating lymphocyte populations on total event plots of (1) FSC-H by FSC-W, and (2) SSC-H by SSC-W. The intersection of these two populations was then plotted on FSC-A by SSC-A axes, and gated again to exclude outliers. The resulting population was typically gated on CD4+/Foxp3+ or CD8+ to identify major T lymphocyte subsets, with more refined gating strategies shown in each figure.

☒ Tick this box to confirm that a figure exemplifying the gating strategy is provided in the Supplementary Information.
